# Sex differences during development in cortical temporal processing and event related potentials in wild-type and fragile X syndrome model mice

**DOI:** 10.1186/s11689-024-09539-8

**Published:** 2024-05-08

**Authors:** Katilynne Croom, Jeffrey A. Rumschlag, Michael A. Erickson, Devin Binder, Khaleel A. Razak

**Affiliations:** 1 Graduate Neuroscience Program, University of California, Riverside, USA; 2 Department of Otolaryngology-Head and Neck Surgery, Medical University of South Carolina, Charleston, USA; 3 Department of Psychology, University of California, 900 University Avenue, Riverside, USA; 4 Biomedical Sciences, School of Medicine, University of California, Riverside, USA

**Keywords:** Autism Spectrum Disorders, Sensory Processing Disorders, Hypersensitivity, Language Impairments, Temporal Processing, Frontal Cortex, Neurodevelopment

## Abstract

**Background:**

Autism spectrum disorder (ASD) is currently diagnosed in approximately 1 in 44 children in the United States, based on a wide array of symptoms, including sensory dysfunction and abnormal language development. Boys are diagnosed ~ 3.8 times more frequently than girls. Auditory temporal processing is crucial for speech recognition and language development. Abnormal development of temporal processing may account for ASD language impairments. Sex differences in the development of temporal processing may underlie the differences in language outcomes in male and female children with ASD. To understand mechanisms of potential sex differences in temporal processing requires a preclinical model. However, there are no studies that have addressed sex differences in temporal processing across development in any animal model of ASD.

**Methods:**

To fill this major gap, we compared the development of auditory temporal processing in male and female wildtype (WT) and *Fmr1* knock-out (KO) mice, a model of Fragile X Syndrome (FXS), a leading genetic cause of ASD-associated behaviors. Using epidural screw electrodes, we recorded auditory event related potentials (ERP) and auditory temporal processing with a gap-in-noise auditory steady state response (ASSR) paradigm at young (postnatal (p)21 and p30) and adult (p60) ages from both auditory and frontal cortices of awake, freely moving mice.

**Results:**

The results show that ERP amplitudes were enhanced in both sexes of *Fmr1* KO mice across development compared to WT counterparts, with greater enhancement in adult female than adult male KO mice. Gap-ASSR deficits were seen in the frontal, but not auditory, cortex in early development (p21) in female KO mice. Unlike male KO mice, female KO mice show WT-like temporal processing at p30. There were no temporal processing deficits in the adult mice of both sexes.

**Conclusions:**

These results show a sex difference in the developmental trajectories of temporal processing and hypersensitive responses in *Fmr1* KO mice. Male KO mice show slower maturation of temporal processing than females. Female KO mice show stronger hypersensitive responses than males later in development. The differences in maturation rates of temporal processing and hypersensitive responses during various critical periods of development may lead to sex differences in language function, arousal and anxiety in FXS.

**Supplementary Information:**

The online version contains supplementary material available at 10.1186/s11689-024-09539-8.

## Background/Introduction

Abnormal sensory processing and delayed language development are core symptoms of ASD [1–5]. This spectrum of disorders has traditionally been diagnosed within the first three years of life, when differences from age-matched typically developing children start to become apparent, particularly with sensory issues and language development [6]. Deficits in sensory processing have been reported in up to 87% of patients and correlate with autism-related social difficulties [7–11]. A sex bias in ASD diagnosis is well established, with the male:female ratio of diagnosis being ~ 3.8:1 [12]. Sex differences seen in the maturation rate of language function in typically developing children [13–18] are further enhanced in children with ASD with males showing slower development and/or more impairments [19–21]. While multiple studies have suggested a link between fetal or early postnatal sex hormone levels and language development, the developmental trajectory and mechanisms of this sex difference are not well understood [22–28].

Mutations in the *Fmr1* (Fragile X Messenger Ribonucleoprotein) gene show a strong link and comorbidity with ASD. The silencing of *Fmr1* results in a loss of the Fragile X Messenger Ribonucleoprotein (FMRP) and Fragile X Syndrome (FXS) [29, 30]. The loss of FMRP causes altered synaptic development and brain plasticity, intellectual deficits, and behaviors related to ASD, including repetitive behaviors, sensory, cognitive, and social impairments [31–36]. Individuals with FXS show abnormal sensory sensitivity and speech and language impairments [1, 35, 37–45]. As FXS is an X-linked disorder, a strong sex bias is present with ~ 1 in 4000 males and ~ 1 in 7000 females affected [46]. Males with FXS are on average more impaired in language development than females, but the developmental mechanisms of sex-differences in language function in FXS are unclear [37, 47–49].

Abnormal development of auditory temporal processing may underlie impaired language function. Auditory temporal modulation cues aid speech recognition [50, 51], and humans’ ability to discriminate temporal cues in sounds is present at a very young age [52, 53]. The inability to process rapidly changing acoustic input during development may interfere with speech perception and phonological processing and may result in language disorders [54]. Individuals with ASD show deficits in detecting sound duration, onset and offset, and rapid spectrotemporal changes [55–59]. Issues with reproducing auditory stimuli lengths are evident in children with ASD, and both children and adults with ASD display atypical neural responses to pitch fluctuations in sequential, repeated auditory stimuli [60–62]. Increased gap-detection thresholds, commonly used to evaluate auditory temporal processing, are seen in ASD. Importantly, children with poorer gap detection scores were also associated with lower phonological processing scores [2]. These studies provide evidence that deficiencies in auditory temporal processing may influence atypical language function in ASD.

The *Fmr1* KO mouse, an animal model of FXS, exhibits abnormal sensory responses similar to humans, providing a useful platform for studying the developmental patterns and neural mechanisms of sensory circuit dysfunction [63]. However, very little is known in terms of sex differences in sensory responses in the *Fmr1* KO mice, or in humans with FXS. Indeed, very little is known about sex differences in the developmental trajectory of sensory responses in any non-human species, including mice of any genotype. To fill this major gap, we recorded sensory electrophysiological responses in female *Fmr1* KO mice across development in this study and compared the responses to previously published data from male *Fmr1* KO mice using identical methods and ages [64]. We tested the hypothesis that sex differences in sensory deficits such as auditory cortical temporal processing and auditory sensitivity emerge in *Fmr1* KO mice from early developmental stages.

We acquired EEG signals from the auditory and frontal cortex (AC, FC) in both *Fmr1* KO and wildtype (WT) mice at three different ages: p21, p30, and p60. A 40 Hz gap-in-noise ASSR (auditory steady state response, hereinafter referred to as gap-ASSR) paradigm was used to measure the cortex's reliability in phase locking to brief gaps in noise at varying modulation depths to assess temporal processing acuity [65]. Gap stimuli have been used extensively to evaluate auditory temporal precision, and EEG recordings are more readily executed in humans compared to single-unit recordings, and thereby facilitate translational relevance [66, 67]. The gap-ASSR paradigm requires the neural generators to synchronize responses to gaps of different widths in noise, providing an objective measure of temporal processing across genotypes and age groups. Auditory event related potentials (ERPs) are consistently of larger amplitudes in humans with FXS, but potential sex differences in FXS are not known. Therefore, we recorded auditory ERPs in mice to examine possible sex differences in hypersensitive responses during development in the AC and the FC. Our data show earlier maturation of temporal processing in *Fmr1* KO female mice compared to male mice, and a larger enhancement of ERP amplitudes in KO female than male mice across development, compared to WT mice.

## Methods

The following age ranges and sample sizes were used in this study: WT-Females: p21 (*n* = 11), p30 (*n* = 9), p60 (*n* = 8) and *Fmr1* KO-Females: p21 (*n* = 8), p30 (*n* = 9), p60 (*n* = 8)]. The data collected on females were compared to previously published WT and *Fmr1* KO male data [64]. None of the female data, or sex comparisons, have been previously published.

All procedures were approved by the Institutional Animal Care and Use Committee at the University of California, Riverside. Mice were obtained from an in-house breeding colony that originated from Jackson Laboratory (Bar Harbor, ME). The mice used for the study are sighted FVB wild-type (Jax, stock# 004828; WT) and sighted FVB *Fmr1* knock-out (Jax, stock# 004624; *Fmr1* KO). This background strain was chosen because our prior developmental work examining cortical parvalbumin and perineuronal nets as well as single unit responses in the auditory cortex and the inferior colliculus have utilized this same strain [68, 69]. One to five mice were housed in each cage under a 12:12-h light–dark cycle and fed ad libitum. A cross-sectional, as opposed to a longitudinal, design was used in this study as it is impractical to place epidural screw electrodes in brains that are still growing.

The ages selected for the sex difference comparison were based on previous findings. Decreased PNN expression surrounding parvalbumin-positive interneurons and cortical hyperexcitability are observed in *Fmr1* KO mice at p21 [70]. Additionally, the p14-21 age corresponds to the critical period for responses to simple tones and maturation of tonotopic maps in the auditory cortex [71, 72]. P30 was chosen because response selectivity to complex sounds has not matured in the auditory cortex until this age [73]. We chose the p60 age group to represent young adulthood. Our previous study in males also showed significant genotype differences in temporal processing at p21 and p30, and normalization at p60. Here we compared developmental trajectories of male and female *Fmr1* KO mice.

### Surgery

Different groups of mice underwent epidural electrode implant surgery at three developmental timepoints: p18-20, p27-p29, p57-p66. Surgical procedures have been previously published [64, 65, 74]. Briefly, mice were anesthetized using intraperitoneal (i.p.) injections of either 80/20 mg/kg of ketamine/xylazine (young mice) or 80/10/1 mg/kg ketamine/xylazine/acepromazine (adult mice). The anesthetic state was monitored closely throughout the procedure by toe pinch reflex every 10–15 min. ETHIQA-XR (1-shot buprenorphine, 3.25 mg/kg body weight) was administered via subcutaneous injection prior to surgery. An incision was made to expose the scalp following the removal of fur and sterilization (alcohol and iodine wipes) of the scalp. A Foredom dental drill was used to drill ~ 1 mm diameter holes in the skull over the right AC, right FC, and left occipital cortex. The screw positions were determined using skull landmarks and coordinates previously reported [65, 74–76]. The wires extending from three-channel posts were wrapped around 1 mm screws and driven into the pre-drilled holes. Dental cement was applied to secure the implant. Mice were placed on a heating pad until fully awake and were allowed 48–72 h for recovery before EEG recordings were made.

### EEG recordings

All EEG recordings were obtained from awake and freely moving female mice, using methods identical to those published for male mice [64]. EEG recordings were performed at three developmental time points: p20-23, p29-31, p59-p70, which we refer to as p21, p30 and p60, respectively. Recordings were obtained from the AC and FC electrodes, using the occipital screw as reference. All recordings were obtained inside a sound-insulated and anechoic booth (Gretch-Ken, OR). Mice were briefly anesthetized with isoflurane and connected to an EEG cable via the implant. Mice were habituated to the recording arena with no stimuli prior to sound evoked recordings. The attached cable was connected via a commutator to a TDT (Tucker Davis Technologies, FL) RA4LI/RA4PA headstage/pre-amp, which was connected to a TDT RZ6 multi-I/O processor. OpenEx (TDT) was used to simultaneously record EEG signals and operate the LED light used to synchronize the video and waveform data. TTL pulses were utilized to mark stimulus onsets on a separate channel in the collected EEG data. The EEG signals were recorded at a sampling rate of 24.414 kHz and down-sampled to 1024 Hz for analysis. All raw EEG recordings were visually examined prior to analysis for artifacts, including loss of signal or signs of clipping. No EEG data were rejected after examination.

### Auditory ERP

Narrowband noise stimuli (6–12 kHz bandwidth, 120 repetitions, 100 ms duration, 5 ms rise/fall time, 0.25 Hz repetition rate) were presented at 75 dB SPL using a speaker (MF1, Tucker Davis Technologies, FL) situated 20 cm above the floor of the arena. ERP analysis and statistics have been previously described [64, 65, 74]. Briefly, the EEG trace was split into epochs using the TTL pulses to mark sound onset. Each trial was baseline corrected, such that the mean of the 250 ms baseline period prior to sound onset was subtracted from the trial trace for each trial. Each trial was then detrended (MATLAB detrend function) and all trials were averaged together.

### Gap-ASSR

The stimulus used to assess auditory temporal processing is termed the ‘40 Hz gaps-in-noise ASSR’ (auditory steady state response, henceforth, ‘gap-ASSR’) [65]. The stimulus contains alternating 250 ms segments of noise and gap interrupted noise presented at 75 dB SPL. The gaps are placed 25 ms apart, resulting in a presentation rate of 40 Hz, a rate that produces the strongest ASSR signal when measured from the AC and frontal regions [77–83]. For each gap-in-noise segment, the gap width and modulation depth are chosen at random. Gaps of 2, 4, 6, 8, 10, or 12 ms widths and modulation depths of 75 and 100% were used. To measure the ability of the cortex to consistently respond to the gaps in noise, inter-trial phase clustering (ITPC) at 40 Hz was measured [84]. The ITPC is based on the distribution of phase angles in the EEG response at 40 Hz (because the stimulus is a 40 Hz train) across all trials and reflects the precise timing of 40 Hz activity in the underlying neural generators. ITPC can be interpreted independently of power. ITPC ranges between 0 and 1, with 0 indicating high variability (uniform distribution) of phase angles across trials, and 1 indicating the same phase angle for every trial. Because ITPC is sensitive to temporal jitter of responses from one trial to the next, this is a useful measure of temporal reliability of responses. The EEG trace was transformed using a dynamic complex Morlet wavelet transform. The trials corresponding to each parametric pair (gap duration + modulation depth) were grouped together. The ITPC was calculated for each time–frequency point as the average vector for each of the phase unit vectors recorded across trials (trial count > 100 trials per parametric pair). The ITPC values at 40 Hz were averaged to extract the mean ITPC for the parametric pairs in the AC and FC.

### Statistics

Statistics were performed on GraphPad Prism (ERP) or R (gap-ASSR). To evaluate the effects of genotype (2 levels) and age (3 levels), or sex (2 levels) and age (3 levels), two-way ANOVA was used for ERP analysis. Post hoc comparisons were carried out with Tukey’s and Bonferroni’s multiple comparisons test. ERP data were tested for normality using Shapiro–Wilk tests. A three-way repeated measures ANOVA was used for the female development gap-ASSR analysis, with the three factors being genotype (2 levels) X age (3 levels) X gap duration (6 levels). Mauchly Tests for Sphericity were utilized and corrected for using the Greenhouse–Geisser corrections if necessary. A two-way repeated measures ANOVA was used for within-genotype sex comparisons at each age, with the two factors being sex (2 levels) X gap duration (6 levels). The Geisser and Greenhouse epsilon hat method was utilized to correct *p*-values for lack of sphericity using the Greenhouse–Geisser corrections if necessary. A repeated measures ANOVA was chosen as multiple gap duration data points were collected from a single mouse in a recording session. Post hoc contrasts with Sidak corrections for multiple comparisons were used. Cortical regions (AC, FC) and modulation depths (75%, 100%) were analyzed separately. Data were evaluated to ensure ANOVA assumptions were met, in particular the assumption of the normality of the residuals. None of the residuals had measures of skewness or kurtosis that exceeded ± 2, which is one indication of acceptable normality [85]. Moreover, the residuals were evaluated via quantile–quantile plots. In each of the analyses, the correspondence between the theoretical normal distribution and the obtained residuals was within acceptable bounds.

## Results

The main goals of this study were to record the developmental trajectory of auditory temporal processing and ERPs in female WT and *Fmr1* KO mouse auditory and frontal cortex and compare these data with previously published data from male counterparts [64]. The aim was to determine whether sex differences in evoked responses were present in WT and KO mice throughout development.

### Delayed development of temporal processing in the FC of female *Fmr1* KO mice

**Fig. 1:**
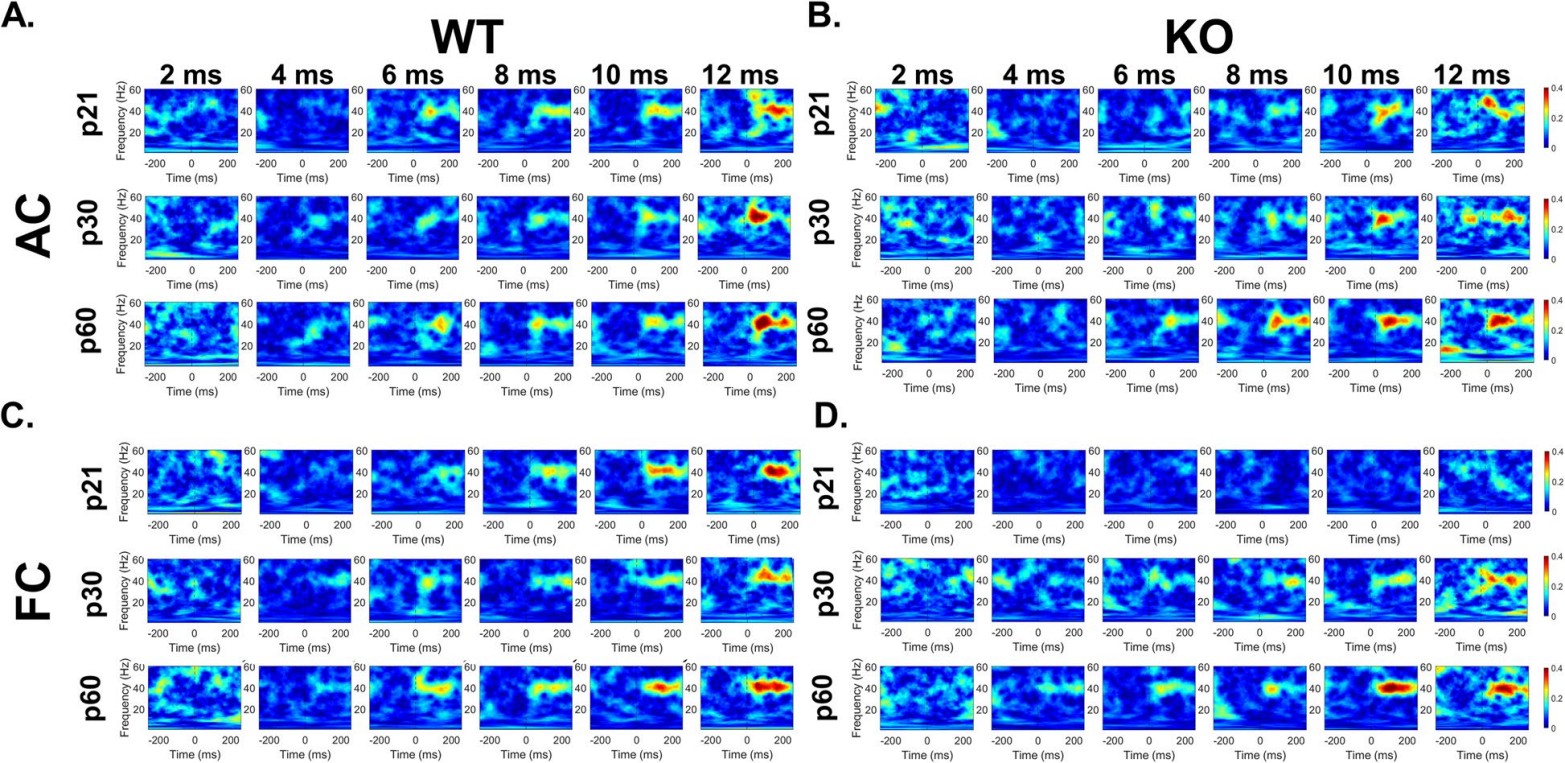
Delayed development of temporal processing in the frontal cortex of female *Fmr1* KO mice. Individual example heatmaps of ITPC generated at 40 Hz at multiple gap durations in p21, p30, and p60 WT (**A**: AC, **C**: FC) and *Fmr1* KO (**B**: AC, **D**: FC) female mice. Qualitative observations of these examples show deficits in cortical temporal processing at p21, but not p30 or p60, in the FC KO mice. No deficits are seen in the AC at any age. All panels show 100% modulation depth. The onset of the gap-ASSR stimulus is at 0 ms in each panel

Auditory temporal processing was assessed using a 40 Hz gap-in-noise ASSR stimulus to determine the ability of auditory and frontal cortex (AC, FC) to consistently phase lock to brief gaps in noise. Manipulating the duration and modulation depth of the gaps allows for the identification of differences in temporal acuity between WT and KO mice and to track developmental changes. Figure 1 shows inter-trial phase clustering (ITPC) heat maps in the 40 Hz gap-ASSR from example female WT (Fig. 1A, C) and *Fmr1* KO (Fig. 1B, D) mice (modulation depth of 100%). The y-axis of each panel shows the ITPC at a specific gap generated with the 40 Hz signal, with increasing gaps across columns. Each row marks a different age. In each panel, zero (faint vertical dashed line) on the x-axis marks the onset of the gap ASSR stimulus. The expected ITPC is at 40 Hz because the stimulus is a 40 Hz train. Therefore, the warm colors indicating higher ITPC (see ITPC scale at the right of Fig. 1) are seen at 40 Hz. Cooler colors indicate relatively low ITPC and are mostly seen for very short gaps, KO mice, and at spectral bands outside 40 Hz.

As expected, both AC and FC are better able to synchronize their responses to longer gaps compared to short gaps (left to right in each row). Across genotypes, there are no qualitative ITPC differences in the AC throughout development (Fig. 1A, B). However, in the FC, deficits are clearly seen at p21, with the *Fmr1* KO mice ITPC barely emerging above background at 40 Hz (Fig. 1C, D). Genotype differences were not observed at p30 or p60 in female mouse AC and FC.

**Fig. 2:**
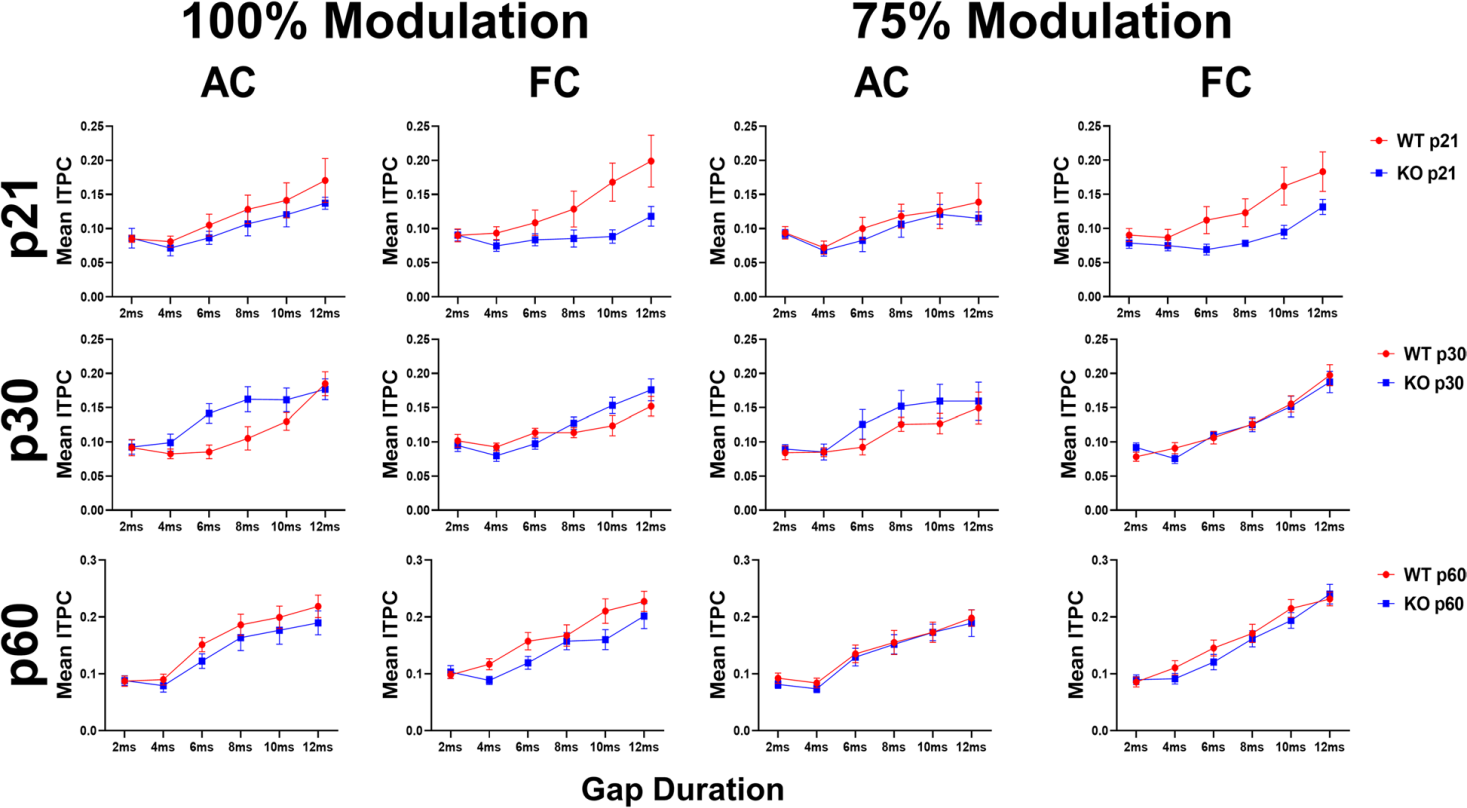
Population analysis shows temporal processing deficits in the FC during development in *Fmr1* KO female mice. Each plot represents the group average ITPC values. Each row represents a different age group: p21 (top), p30 (middle), and p60 (bottom). The left columns represent AC and FC data at 100% modulation depth, and the right columns represent AC and FC data at 75% modulation depth. *Fmr1* KO female mice show significant deficits in the FC, but not the AC, at p21. No genotype differences are seen at p30 or p60. Full data results are shown in Additional File 1

**Fig. 3:**
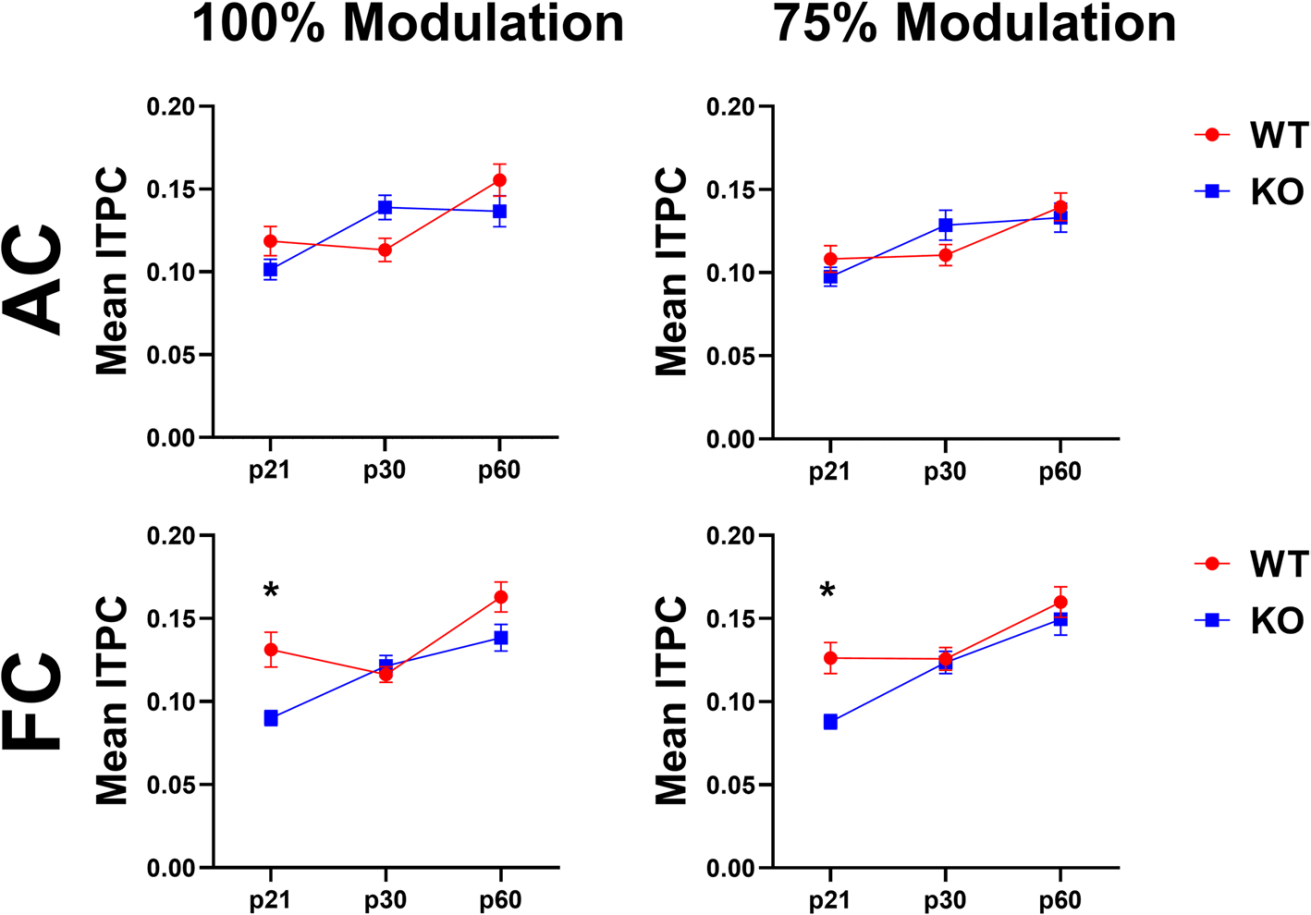
Developmental delay in auditory temporal processing in the FC of female *Fmr1* KO mice. Each plot represents the group average ITPC values collapsed across gap widths. Columns represent different modulation depths, and rows represent different cortical regions (Columns – left = 100% modulation, right = 75% modulation; Rows – top = AC, bottom = FC). KO mice show a significant ITPC deficit only at p21 in the FC at both modulation depths, but not at p60. A genotype difference was not seen at any age or modulation depth in the AC

Quantitative analyses across the population of female mice recorded support these suggestions (Fig. 2 and Additional File 1). Statistical analyses using gap duration, age and genotype as factors show a main effect of gap duration in the AC and FC. This is not surprising as the cortex responds with more consistent phase angles (less temporal jitter) across trials to the 40 Hz stimulus with longer gap durations. No genotype differences were identified at any age or modulation depth in the AC, similar to our previously published male data (Fig. 2, Additional File 1) [64]. Figure 3 shows average ITPCs collapsed across all the gap durations. In the AC, there are no statistical differences between WT and KO mice at any age. Taken together, these data suggest developmental improvement in temporal processing of female mice, but no effects of the loss of FMRP, in the AC at any age.

In the frontal cortex, however, significant genotype effects were seen (Fig. 2, Additional File 1). ITPC increased with age in both WT and *Fmr1* KO females. At both modulation depths, our results show a significant reduction of ITPC in female KO mice at p21 compared to WT female mice (75% MD – *p* = 0.0068; 100% MD – *p* = 0.0026). This deficit is not present at p30 or p60 (p30 – 75% MD *p* = 1.000, 100% MD *p* = 1.000; p60 – 75% MD *p* = 0.9973, 100% MD *p* = 0.3909). Evidence of a developmental delay is shown more directly by collapsing across gaps, as no significant genotype effect can be seen in the FC at p30 or p60, but a significant reduction is seen in the KO females at P21 (Fig. 3). Overall, these data show improvement in phase locking to gap-ASSR stimuli during development in both AC and FC in both genotypes, but there is a FC-specific delay in temporal processing in female *Fmr1* KO mice.

### Temporal processing matures faster in *Fmr1* KO females than males

**Fig. 4:**
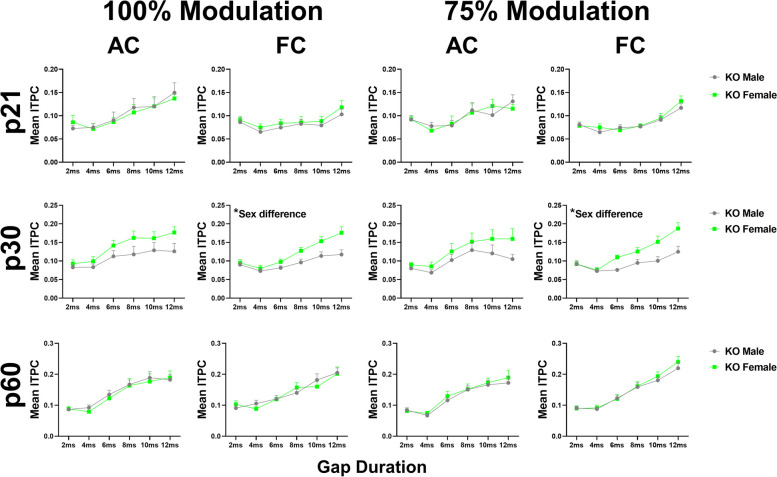
Temporal processing matures faster in *Fmr1* KO females than males. Each plot represents the group average ITPC values. Each row represents a different age group: p21 (top), p30 (middle), and p60 (bottom). The left columns represent AC and FC data at 100% modulation depth, and the right columns represent AC and FC data at 75% modulation depth. No significant sex difference in the AC at any modulation depth or gap duration. Female KO mice have significantly higher ITPC in the FC at both modulation depths at p30, but not p21 or p60. Full data results are shown in Additional File 2

**Fig. 5:**
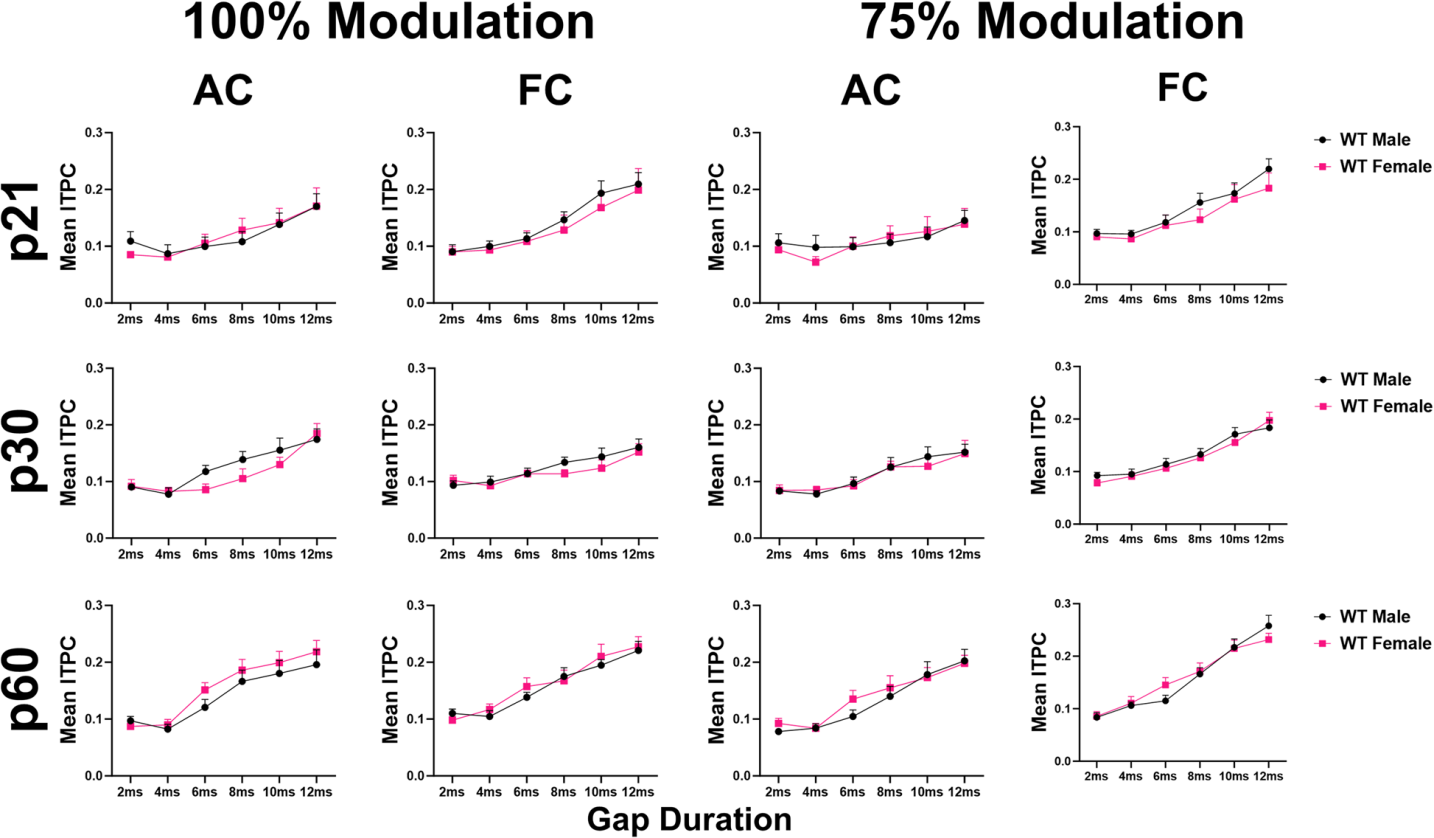
No sex difference in auditory temporal processing in WT mice at any in the AC or FC. Each plot represents the group average ITPC values. Each row represents a different age group: p21 (top), p30 (middle), and p60 (bottom). The left columns represent AC and FC data at 100% modulation depth, and the right columns represent AC and FC data at 75% modulation depth. No significant sex difference in the AC or FC at any modulation depth or gap duration. Full data results are shown in Additional File 3

Figure 4 compares *Fmr1* KO female and male data [64]. The results show no significant sex difference in the AC at any modulation depth or gap duration. There is a sex difference in the FC at both modulation depths at p30 (p30: 100% MD – *p* = 0.0160; 75% MD – *p* = 0.0034), with female KO mice having significantly higher ITPC compared to males, suggesting that female KO mice have more consistent temporal responses than males across trials at this age. There is no sex difference in *Fmr1* KO mice at p21 or p60. These results show a faster maturation of auditory temporal processing in the FC of KO females compared to males. We also compared male and female WT gap-ASSR responses to test whether this sex difference is unique to *Fmr1* KO mice or is a normative pattern (Fig. 5). No sex difference was seen in WT mice at any modulation depth in either cortical region. This confirms that female *Fmr1* KO mice show improved auditory temporal processing by p30, while males do not reach WT levels until after p30. Full statistical analysis for WT and KO sex difference gap-ASSR analyses can be found in Additional Files 2 and 3.

### Female *Fmr1* KO mice show enhanced cortical ERP amplitudes across development

**Fig. 6:**
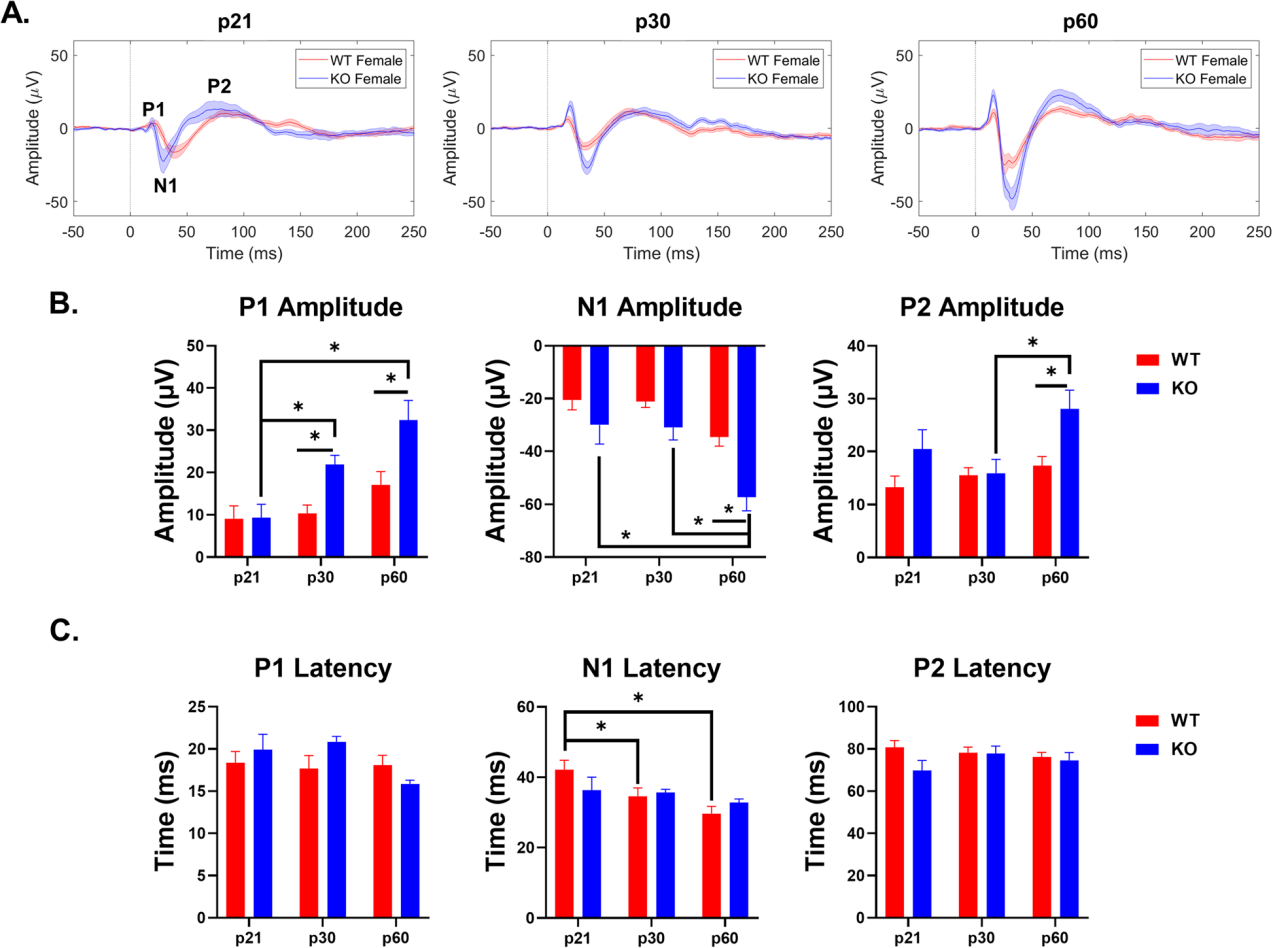
Age and genotype impact ERP amplitudes and latencies in the AC of female mice. (**A**) Average ERPs recorded in the AC for WT and KO female mice at p21 (left), p30 (middle), and p60 (right). (**B**) Population averages of AC ERP wave amplitudes. P1 amplitude significantly increases in KO mice with development, but not WT mice. KO mice have increased P1 amplitudes compared to WT at p30 and p60. N1 and P2 amplitudes are enhanced in adult KO females compared to WT and increase with age. (**C**) AC ERP wave latencies. N1 latency decreases with age in WT mice. Full data results are shown in Additional File 4

**Fig. 7:**
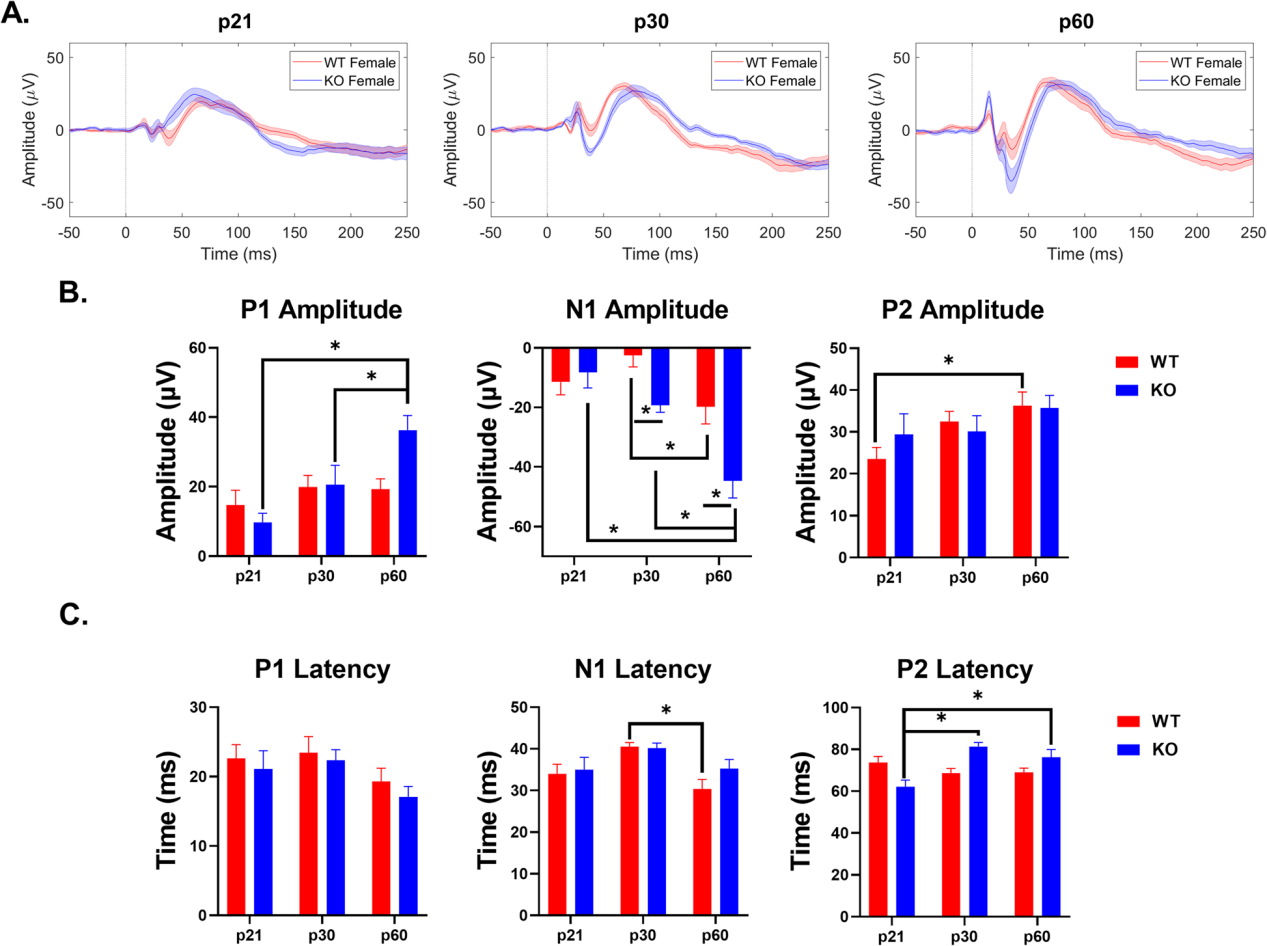
Age and genotype impact ERP amplitudes and latencies in the FC of female mice. (**A**) Average ERPs recorded in the FC for WT and KO female mice at p21 (left), p30 (middle), and p60 (right). (**B**) Population averages of FC ERP wave amplitudes. P1 amplitude significantly increases in KO mice with development, but not WT mice. N1 amplitudes are enhanced in KO females at p30 and p60. N1 amplitudes increase with age in WT and KO females. P2 amplitudes increase with age in WT mice. (**C**) FC ERP wave latencies. N1 latency decreases with age in WT mice. P2 latency fluctuates with age in KO mice. Full data results are shown in Additional File 4

ERPs consist of a series of voltage fluctuations, referred to as ‘waves’ (P1, N1, P2). These waves are evoked at specific latencies after sound onset and are associated with the population activity in specific brain regions. Measuring the amplitudes and latencies of these waves allow for the assessment of neuronal response synchrony or hypersensitivity to sound presentation. Additional File 4 and Figs. 6 and 7 show the complete ANOVA results of female WT and *Fmr1* KO ERP data across development and genotypes.

#### Auditory Cortex ERP

All three peaks (P1, N1 and P2) show a larger increase with age in the *Fmr1* KO female mice compared to WT females, resulting in significant age-dependent ERP amplitude differences (Fig. 6A). ERP P1 amplitude increases with age in female *Fmr1* KO mice (interaction effect: *p* = 0.0507; main effect of age: *p* < 0.0001; KO p21-30: *p* = 0.0202; KO p21-60: *p* < 0.0001). P1 amplitude is also significantly increased in KO females compared to WT at p30 and p60 (main effect of genotype: *p* = 0.0010; p30: *p* = 0.0341; p60: *p* = 0.0054) (Fig. 6B). N1 amplitude increases with age in KO mice, but only shows a significant genotype effect at p60 (main effect of age: *p* < 0.001; KO p21-60: *p* = 0.0006; KO p30-60: *p* = 0.0006; main effect of genotype: *p* = 0.0005; p60: *p* = 0.0048) (Fig. 6B). Similarly, P2 amplitude increased with age in KO mice and was significantly elevated compared to WT at p60 (main effect of age: *p* = 0.0257; KO p30-60: *p* = 0.0058; main effect of genotype: *p* = 0.0059; p60: *p* = 0.0226) (Fig. 6B). No genotype or age differences were seen in P1 or P2 latencies, but N1 latency decreased with age in WT females (main effect of age: 0.0059; WT p21-30: *p* = 0.0504; WT p21-60: *p* = 0.0010) (Fig. 6C). These data show increased ERP amplitudes in the AC of female *Fmr1* KO mice as observed consistently in humans with FXS. Furthermore, this hypersensitivity increases with age in female KO mice.

#### Frontal Cortex ERP

As in the AC, frontal cortex ERP amplitudes show a more pronounced developmental increase in female KO mice, compared to WT females (Fig. 7A). ERP P1 and N1 amplitudes increase with age in KO female mice (P1 – interaction effect: *p* = 0.0318; main effect of age: *p* = 0.0021; KO p21-p60: *p* = 0.0002; KO p30-p60: *p* = 0.0277; N1 – interaction effect: 0.0109; main effect of age: *p* < 0.0001; KO p21-60: *p* < 0.0001; KO p30-p60: *p* = 0.0011) (Fig. 7B). N1 and P2 amplitudes increase with age in WT female mice (N1 – main effect of age: *p* < 0.0001; WT p30-p60: *p* = 0.0314; P2 – main effect of age: *p* = 0.0283; WT p21-60: *p* = 0.0249) (Fig. 7B). Female KO mice have increased N1 amplitudes at p30 and p60 compared to WT (main effect of genotype: 0.0014; p30: *p* = 0.0354; p60: *p* = 0.0019) (Fig. 7B). N1 and P2 latencies showed developmental fluctuations in WT and KO mice, respectively (N1 – main effect of age: 0.0020; WT p30-p60: 0.0440; P2 – main effect of age: 0.0365; KO p21-30: *p* < 0.0001; KO p21-60: *p* = 0.0034) (Fig. 7C). These data show increased ERP amplitudes, specifically N1, in the FC of female *Fmr1* KO mice.

### Development of WT and *Fmr1* KO male and female ERP phenotypes

#### Auditory cortex – WT mice

**Fig. 8:**
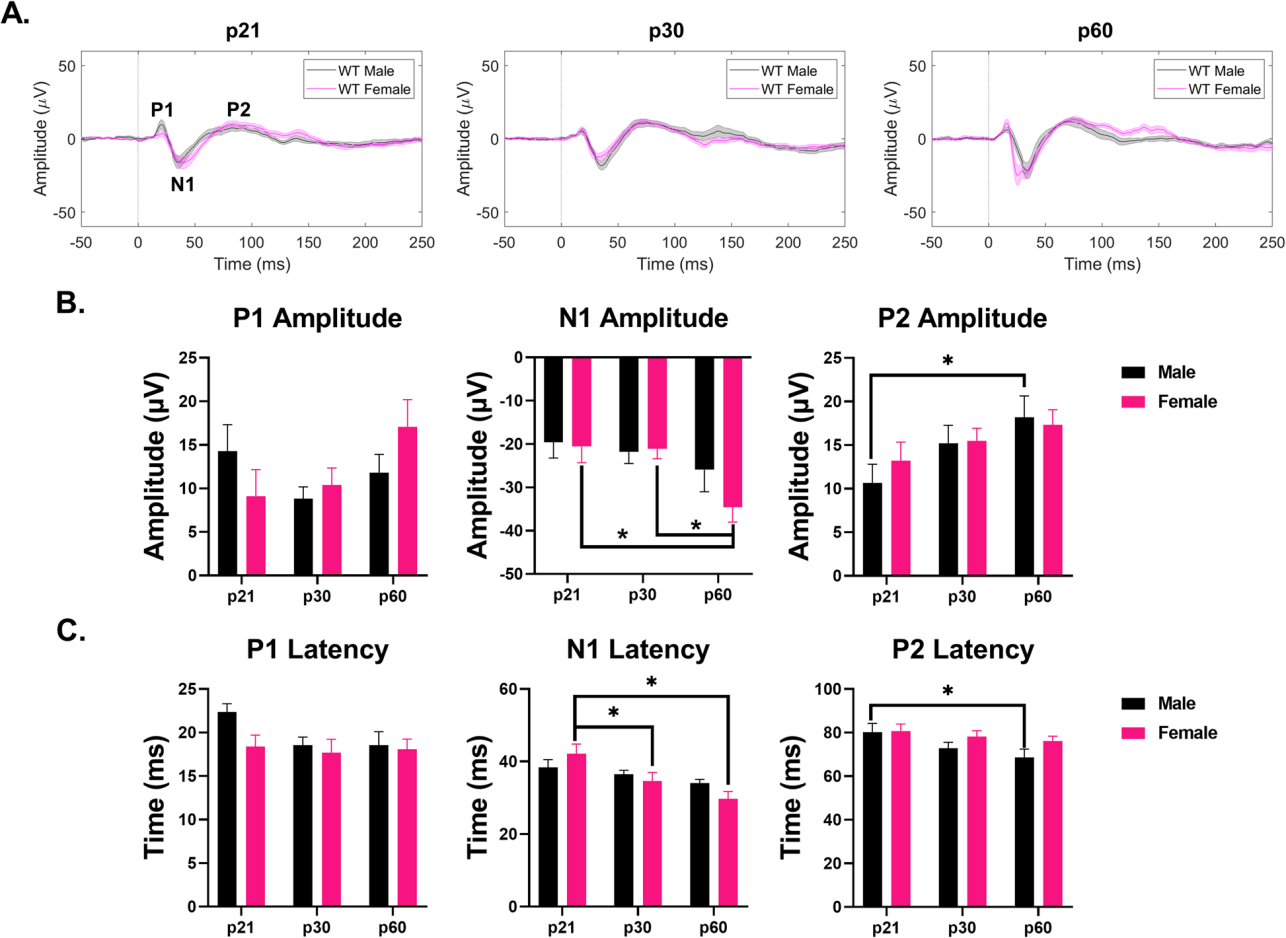
No sex difference in ERP amplitudes or latencies in the AC of WT mice. (**A**) Average ERPs recorded in the AC for WT male and female mice at p21 (left), p30 (middle), and p60 (right). (**B**) Population averages of AC ERP wave amplitudes. N1 amplitudes increase with age in females. P2 amplitudes increase with age in males. (**C**) AC ERP wave latencies. N1 latency decreases with age in females. P2 latency decreases with age in males. Full data results are shown in Additional File 5

ERP P1 amplitudes were not impacted by age or sex in the AC. However, both N1 and P2 amplitudes were affected by age (N1 – main effect of age: *p* = 0.0188; P2 – main effect of age: *p* = 0.0264) (Fig. 8B). Specifically, female and male N1 and P2 amplitudes increased with age, respectively (N1 – female p21-p60: *p* = 0.0330; P2 – male p21-p60: *p* = 0.0309) (Fig. 8B). N1 and P2 latencies were also impacted by age (N1 – main effect of age: *p* = 0.0004; P2 – main effect of age: *p* = 0.0519) (Fig. 8C). N1 latency decreased with age in female mice (p21-p30: *p* = 0.0233; p21-p60: *p* = 0.0002) (Fig. 8C). P2 latency decreased with age in males (p21-p60: *p* = 0.0316) (Fig. 8C). No sex differences were seen in any wave amplitude or latency. Overall, these results suggest that responses are similar in male and female WT mice throughout development in the AC. Additional File 5 shows the complete ANOVA analyses of male and female WT AC ERP data across development.

*Frontal cortex* – *WT mice.*

**Fig. 9:**
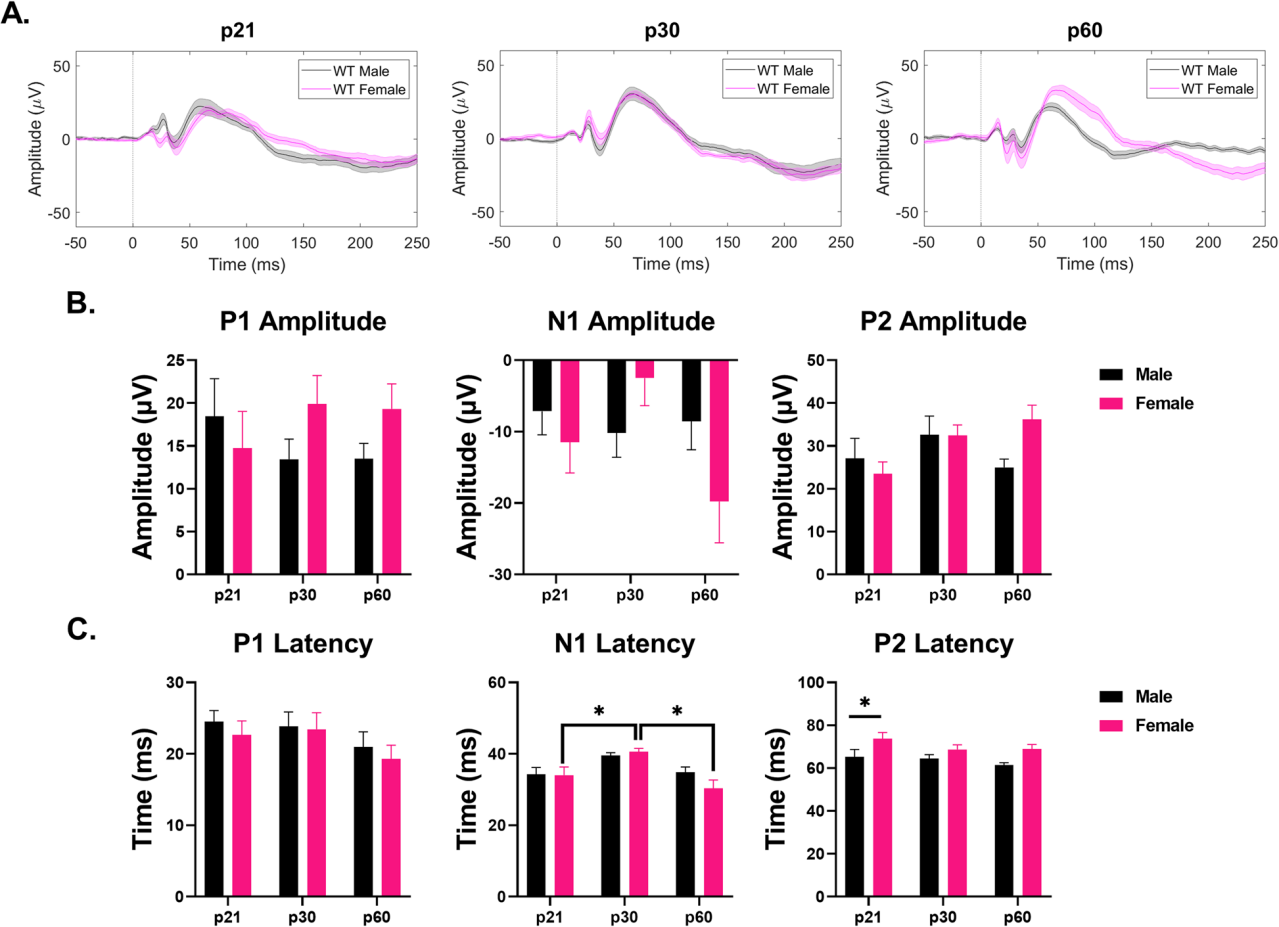
Sex difference in ERP latencies in the FC of WT mice. (**A**) Average ERPs recorded in the FC for WT male and female mice at p21 (left), p30 (middle), and p60 (right). (**B**) Population averages of FC ERP wave amplitudes. No impact of age or sex on any ERP wave amplitude. (**C**) FC ERP wave latencies. N1 latency fluctuates with age in females. P2 latency is increased in female WT mice compared to males. Full data results are shown in Additional File 5

ERP wave amplitudes were not affected by age or sex in the FC (Fig. 9B). N1 latency showed developmental fluctuations in female mice (main effect of age: *p* = 0.0002; p21-p30: *p* = 0.0253; p30-p60: *p* = 0.0009) (Fig. 9C). A significant sex difference was identified in P2 latencies at p21 (main effect of sex: *p* = 0.0011; p21: *p* = 0.0379) (Fig. 9C). These results suggest no significant sex difference in ERP peak amplitudes in the FC of WT mice during development. Additional File 5 shows the complete ANOVA analyses of male and female WT FC ERP data across development.

#### Auditory cortex – *Fmr1* KO mice

**Fig. 10:**
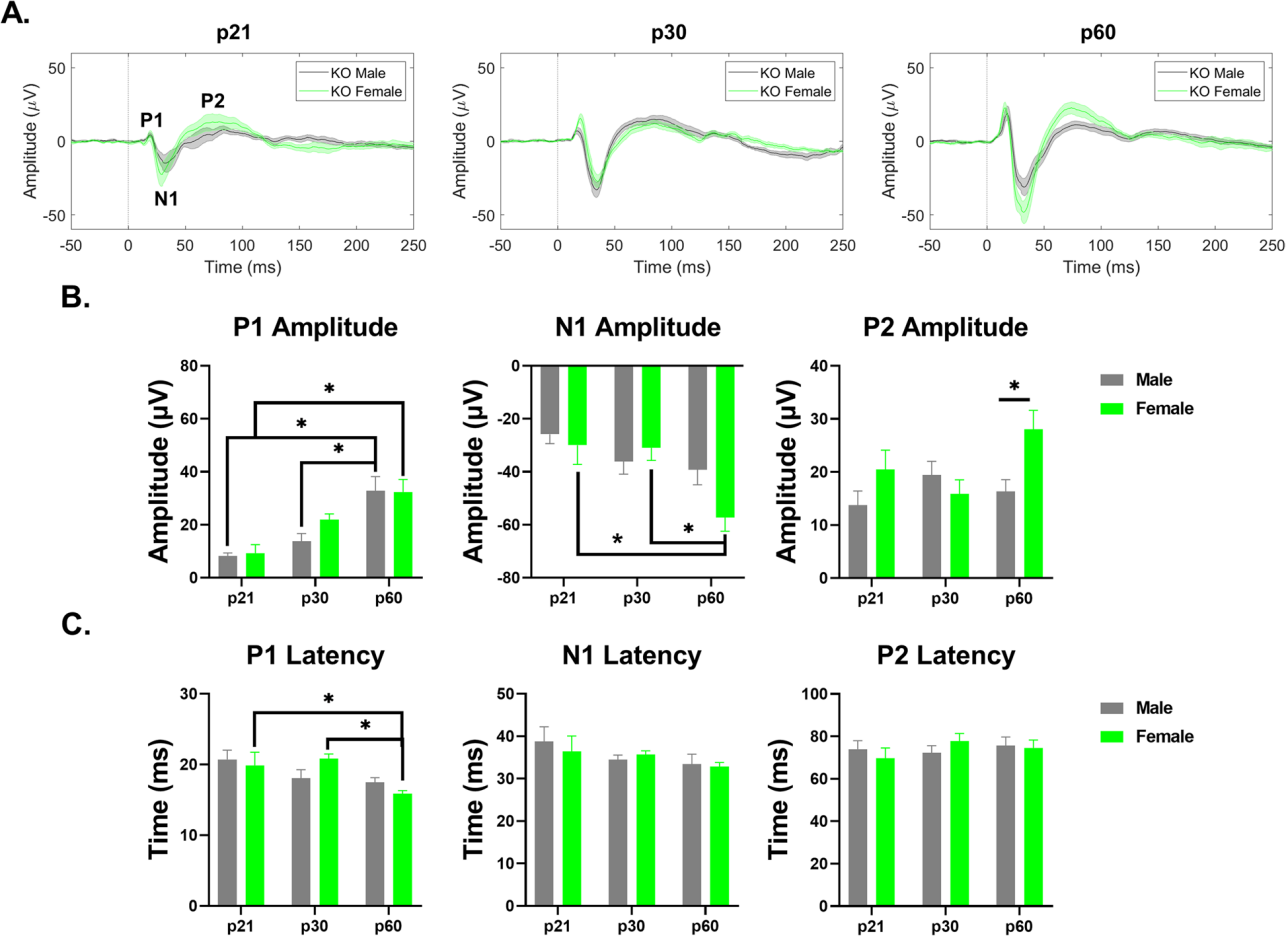
Sex difference in ERP amplitudes in the AC of *Fmr1* KO mice. (**A**) Average ERPs recorded in the AC for *Fmr1* KO male and female mice at p21 (left), p30 (middle), and p60 (right). (**B**) Population averages of AC ERP wave amplitudes. P1 amplitudes increase with age in male and female KO mice. N1 amplitudes increase with age in females. P2 amplitudes are significantly higher in adult female KO mice. (**C**) AC ERP wave latencies. P1 latency decreases with age in KO females. Full data results are shown in Additional File 6

ERP P1 amplitudes increased with age in male and female KO mice (main effect of age: *p* < 0.0001; male – p21-p60: *p* < 0.0001, p30-p60: *p* = 0.0008; female – p21-p60: *p* = 0.0004) (Fig. 10B). N1 amplitude significantly increased with age only in females (main effect of age: *p* = 0.0010; p21-p60: *p* = 0.0036; p30-60: *p* = 0.0039) (Fig. 10B). P2 amplitude was significantly elevated in adult female KO and increased with age (interaction effect: *p* = 0.0285 main effect of sex: *p* = 0.0378; p60: *p* = 0.0156) (Fig. 10B). P1 latency significantly decreased with age in females (main effect of age: *p* = 0.0055; p21-p60: *p* = 0.0532; p30-60: *p* = 0.0106), but no sex or age difference was seen in N1 or P2 latencies (Fig. 10C). The results suggest that adult female *Fmr1* KO mice have increased hypersensitivity, based on ERP amplitudes, compared to adult males in the AC. Additional File 6 shows the complete ANOVA analyses of male and female *Fmr1* KO AC ERP data across development.

#### Frontal cortex – *Fmr1* KO mice

**Fig. 11:**
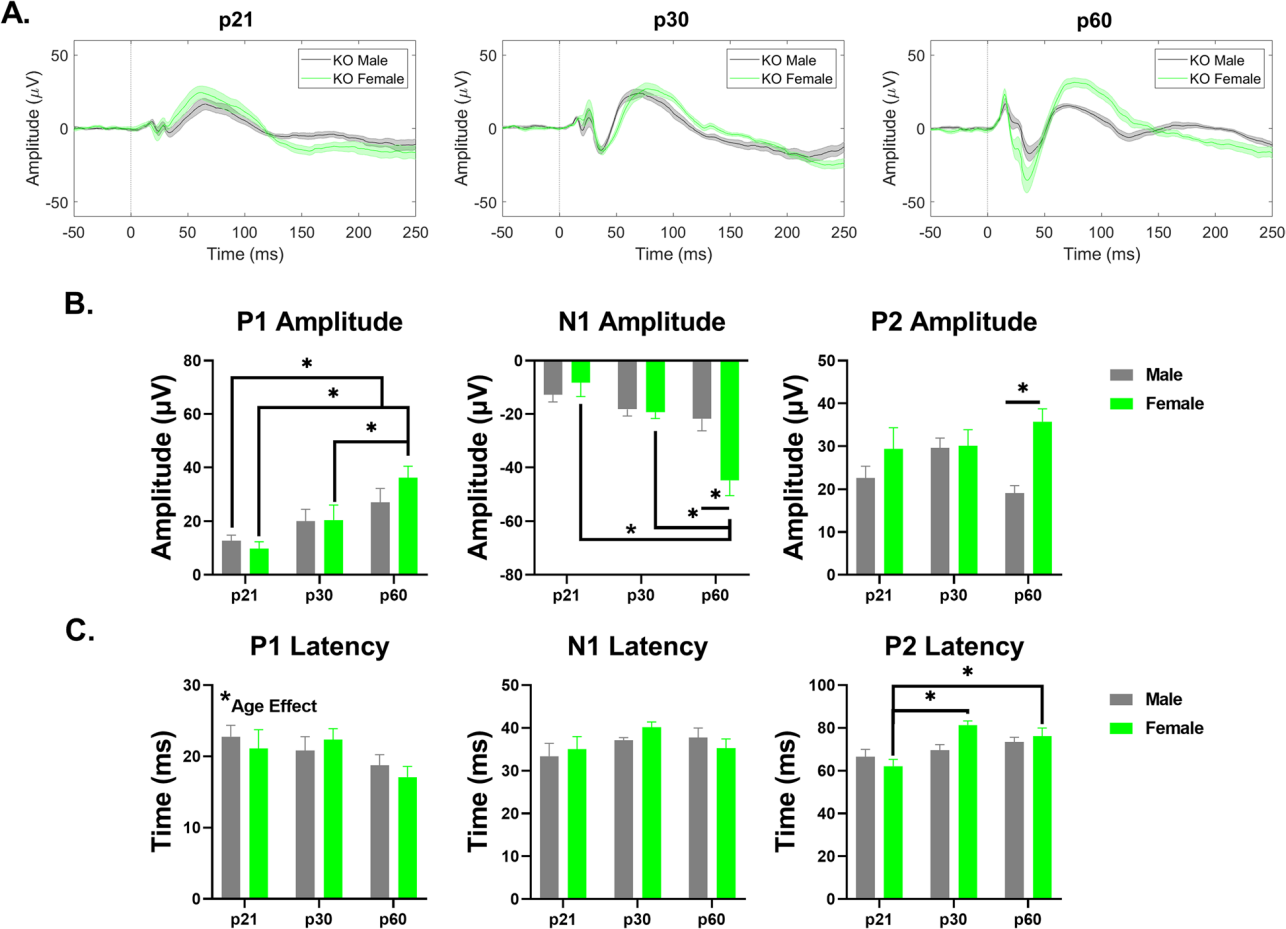
Sex difference in ERP amplitudes in the FC of *Fmr1* KO mice. (**A**) Average ERPs recorded in the FC for *Fmr1* KO male and female mice at p21 (left), p30 (middle), and p60 (right). (**B**) Population averages of FC ERP wave amplitudes. P1 amplitudes increase with age in male and female KO mice. N1 amplitudes increase with age in females. N1 and P2 amplitudes are significantly higher in adult female KO mice. (**C**) FC ERP wave latencies. P1 latency is impacted by age. P2 latency increases with age in KO females. Full data results are shown in Additional File 6

Both P1 and N1 amplitudes increased with age in female KO mice (P1 – main effect of age: *p* = 0.0001; p21-60: *p* = 0.0006; p30-p60: *p* = 0.0475; N1 – main effect of age: *p* < 0.0001; p21-p60: *p* < 0.0001; p30-p60: *p* = 0.0002) (Fig. 11B). However, only P1 amplitudes significantly increased with age in male KO (p21-p60: *p* = 0.0424) (Fig. 11B). Female KO have significantly increased N1 and P2 amplitudes compared to males, which increases age (N1 – interaction effect: *p* = 0.0026; main effect of sex: *p* = 0.0478; p60: *p* = 0.0004; P2 – interaction effect: *p* = 0.0376; main effect of sex: *p* = 0.0027; p60: *p* = 0.0011) (Fig. 11B). Main effects of age were identified in P1 and P2 latencies, with P2 latencies increasing during development in females (P1 – main effect of age: 0.0570; P2 – interaction effect: *p* = 0.0273; main effect of age: *p* = 0.0004; p21-p30: *p* = 0.0001; p21-60: *p* = 0.0068) (Fig. 11C). Overall, these results suggest that ERP amplitudes increase with age in both males and females, but female *Fmr1* KO mice develop increased hypersensitivity with age compared to males in the FC. Additional File 6 shows the complete ANOVA analyses of male and female *Fmr1* KO FC ERP data across development.

## Discussion

The major novel contribution of this study is the identification of sex differences in the developmental trajectories of auditory temporal processing and auditory ERP amplitudes in *Fmr1* KO mice (summary in Table 1). The results show genotype, cortical region and age-specific abnormalities in gap-ASSR responses and ERPs in female mice. A significant developmental delay was seen in gap-ASSR responses in the FC, but not the AC, of female *Fmr1* KO mice compared to WT female mice. However, when compared to male *Fmr1* KO mice, female *Fmr1* KO mice show faster maturation of temporal processing. ERP amplitudes were significantly higher in *Fmr1* KO females than WT females throughout development in the AC and FC, compared to female WT mice. However, adult *Fmr1* KO mice displayed sex differences, with females showing increased N1 and P2 amplitudes compared to males. There were no sex differences in temporal processing or ERP amplitudes in WT mouse cortex indicating that the KO sex differences are not normative, and are related to the loss of FMRP. Taken together, these data show diverging trajectories of ERP and temporal processing phenotypes in female *Fmr1* KO mice, with earlier normalization of temporal processing, but more hypersensitive responses with development, compared to KO males.

**Table 1:** Summary of genotype differences of both sexes in gap-ASSR/ERP measures across development. ‘Yes’ and ‘No’ indicates whether a genotype effect was present between WT and *Fmr1*-KO mice

		**p21**		**p30**		**p60**	
**Stimuli**	**Cortical Region**	Male	Female	Male	Female	Male	Female
**Gap-ASSR**	AC	*NO*	*NO*	*NO*	*NO*	*NO*	*NO*
	FC	*YES*	*YES*	*YES*	*NO*	*NO*	*NO*
**ERP**	AC	*NO*	*NO*	*NO*	*YES*	*YES*	*YES*
	FC	*NO*	*NO*	*NO*	*YES*	*YES*	*YES*

EEG recordings from humans with FXS demonstrate altered cortical oscillatory activity, including elevated broadband gamma power and reduced phase locking to auditory spectrotemporal modulations, particularly ~ 40 Hz. Increased ERP amplitudes are also commonly seen across studies of humans with FXS [86–92]. Sex differences in EEG responses in FXS have not received much attention, however, compared to behavioral studies [93–96]. Ethridge et al. (2019) showed that in the resting state, females with FXS showed increased alpha power relative to typically-developing females, whereas a reduction in alpha power is seen in male FXS patients [86, 97, 98]. In a follow-up study, Smith et al. (2021) show that males with FXS have a lower peak alpha frequency, but not females [99]. Additionally, females with FXS show stronger phase locking to spectrotemporally modulated sounds than FXS males [86]. These findings suggest sex differences in EEG responses in humans with FXS. Our findings of a female advantage in temporal processing in *Fmr1* KO mice is consistent with the human studies.

One major finding of this study is the elevated ERP amplitudes in female *Fmr1* KO mice compared to WT females, and compared to male *Fmr1* KO mice, in both cortical regions. The P1-N1-P2 ERP complex marks the pre-attentive detection of sound and can vary with stimulus features. P1 and N1 amplitudes mark initial sound detection, including thalamocortical input and primary auditory cortex activity, respectively. P2 amplitudes are thought to be related to arousal as auditory input to the mesencephalic reticular activating system contributes to P2 generation [100]. Because N1 and P2 are generated by structures involved in early auditory processing, their enhancement, which is commonly seen in FXS, may reflect altered perception of auditory stimulus [101]. Our data shows that female KO mice have significantly larger P2 amplitudes in both AC and FC compared to male KO mice. This suggests enhanced activation of the arousal component in female *Fmr1* KO mice. Arousal, along with anxiety and avoidance, represent three key behaviors exhibited in response to acute, potential, and sustained threats. Furthermore, these are the typical responses to aversive or dangerous stimuli. Previous studies suggest that the dysregulation of these responses can result in clinical manifestation of emotional disorders, including anxiety and depression [46]. It is possible in humans with FXS that evoked sensory responses are larger in female patients compared to males. Gesi et al., (2021) found that adult females with ASD reported significantly higher scores than men in the hyper/hyporeactivity to sensory input domain, but clearly additional studies are needed to determine if robust sex differences are seen in abnormal sensory sensitivity in humans with ASD, as suggested by our preclinical data [102]. While males with ASD more commonly show externalizing behavior problems, such as aggression, hyperactivity and restricted behaviors, females with FXS show greater internalizing symptoms, including anxiety and depression, as well as social difficulties [103–108]. These opposing symptomologies may be due in part to increased activation of the arousal system in females with FXS. Increased N1 amplitudes were seen in female FC (and a trending increase of N1 in AC), compared to male KO mice suggesting that hypersensitive cortical responses are further enhanced in female mice. No sex differences were seen in WT ERP amplitudes suggesting a deviation in KO females from a normative trajectory. The difference between male and female KO mice in ERP amplitude is largest in the P60 group, suggesting a late developing sex difference in hypersensitivity.

A second major finding of this study is that temporal processing matures faster in the female *Fmr1* KO mice, compared to males. In male KO mice, reduced ITPC is seen in the FC at both p21 and p30, but in the female KO mice, reduced ITPC is only present at p21. While tonotopic maps and the balance between excitatory and inhibitory inputs are established earlier in development (< p21), the p30-40 window is a critical period for development of selectivity for spectrotemporally complex sounds in the mouse auditory cortex [71–73, 109–112]. Impairments in temporal processing during this time window in male *Fmr1* KO mice will lead to abnormal development of cortical selectivity for complex sounds, and consequently to long-term abnormalities in auditory processing. In female *Fmr1* KO mice, temporal processing is WT-like before p30 and this earlier maturation may result in less severe long term consequences in processing of complex sounds. Disruptions of critical period timelines cause long term impairments in behavioral phenotypes. Although these behaviors might appear normalized by adulthood, any irregularities during key developmental phases will have long-term consequences for behaviors that build on normal development of responses. For example, developmental delay in FC temporal processing may lead to long term abnormalities in behaviors that depend on accurate temporal processing such as speech, language and binaural processing. Given the importance of selectivity to spectrotemporal cues in the development of human speech and language function, a similar delay in development of normal temporal processing in males with FXS, compared to females, will result in sex differences in long term deficits in language function. Studies of development of temporal processing and associations with language function are needed in male and female children with FXS.

The development of temporal response properties in the primary auditory cortex of both mice and rats has been shown to be cell-type specific [113, 114]. Although inhibitory responses mature later than excitatory responses, regular-spiking neurons (putative excitatory cells) demonstrate weaker stimulus-following ability compared with fast-spiking (putative inhibitory) neurons [114, 115]. Postsynaptic current duration also differs in the developing auditory cortex, such that inhibitory currents are prolonged compared to excitatory and cause a slower following capacity of two closely timed stimuli [71, 114, 116, 117]. Although the inhibitory duration gradually shortens with development, the longer durations could cause overlap and summation of inhibitory inputs evoked by closely following stimuli, such as in the beginning and end of a gap. Inhibitory dysfunction in FXS is well-established. Nomura et al. (2017) demonstrated a delay in the maturation of the intrinsic properties of fast-spiking interneurons in the sensory cortex as well as a deficit in the formation of excitatory synaptic inputs on to these neurons in *Fmr1* KO mice at p9 [118]. Inhibitory circuits have been implicated in gap detection, however it has been suggested that they provide dynamic gain control over local activity rather than play a specialized role in gap detection. Specifically, Keller et al. (2018) showed that parvalbumin-positive interneurons have stronger on- and off-responses as well as post-response suppression compared to pyramidal neurons. Similar properties were seen for white noise bursts, suggesting that these are generalized response properties of parvalbumin-positive cells [119]. Given the impairment of inhibition in FXS, future studies should investigate the role of inhibitory cell types using gap detection paradigms. While these mechanisms may underlie improved temporal processing with age, there appears to be no sex differences in the WT mice. How sex differences emerge in the *Fmr1* KO mice has not been explored in terms of cell-type specific responses. However, it should be noted that temporal processing deficits may arise from local cortical circuit deficits and/or from subcortical deficits (including brainstem deficits) [69, 120]. FMRP is normally expressed along most of the auditory pathway, and future studies should examine the effects of regional FMRP loss along the auditory pathway on temporal processing development in male and female mice.

A consistent phenotype seen in both male and female *Fmr1* KO mice is that developmental delays in temporal processing are seen in the FC, but not the AC. These findings suggest two key points. Firstly, a lack of deficit in the AC suggests that the FC does not simply inherit auditory responses from the AC, but rather additional local processing within the FC and/or auditory pathways that bypass the AC may be involved in producing phase locked responses in the FC. Secondly, the dichotomy of maturation in males and females could bring about long-term consequences in the FC related to top-down interactions and could possibly give rise to the opposing timelines of language development seen in humans with FXS. The FC induces top-down modulation of AC responses in a task- and attention-dependent manner [121]. FC-AC connection and its modulation of speech have also been evaluated in humans with FXS. Speech production depends on feedforward control and the synchronization of neural oscillations between the FC and AC. Specifically, the interactions of these two regions allow for comparison of the corollary discharge of intended speech generated from an efference copy of speech to the actual speech sounds produced, a process essential for making adaptive adjustments to optimize future speech [122]. Furthermore, top-down corticothalamic projections to the medial geniculate body have been shown to influence temporal processing and stimulus encoding [123]. Atypical regional connectivity patterns, with both hyper- and hypo-connectivity are observed in ASD [124, 125]. Long-range connectivity appears to be reduced, while local connectivity may be increased. The few studies in FXS that have examined cross-regional or cross-frequency coupling show abnormal connectivity. However, even fewer studies have examined sex differences in connectivity. Wang et al. (2017) used both males and females with FXS and found increased theta-to-gamma but decreased alpha-to-gamma band amplitude coupling in resting EEG signals in both sexes [98]. Schmitt et al. (2022) also reported gamma band hyper-connectivity and alpha band hypo-connectivity within frontal cortex in individuals with FXS, but once again found there to be no sex difference [126]. Future studies should examine sex differences in FC-AC functional connectivity during development in humans with FXS to identify potential correlations with abnormal language development.

The mechanisms responsible for the earlier maturation of temporal processing in the female *Fmr1* KO mice are unclear. A recent human study discovered a prolonged alpha state during the pre-stimulus period of an auditory evoked task in females with FXS. Norris et al. (2022) hypothesized that the length of time spent in alpha may reflect a compensatory mechanism that could potentially ‘rescue’ sensory processing abilities [127]. Therefore, the sustained alpha state identified in females could account for improvements seen in females versus males with FXS. Another potential mechanism involves a sex-specific interaction between Group 1 metabotropic receptors (mGluR1 and mGluR5) and estrogen receptor α (ERα). This is an intriguing hypothesis as elevated mGluR5 signaling is heavily reported in FXS [30, 128, 129]. This sex-specific interaction between Group 1 metabotropic receptors and ERα has been identified specifically in female neurons in multiple brain regions [130–133]. In the hippocampus, estradiol acts via ERα to initiate postsynaptic mGluR1-dependent mobilization of the endocannabinoid anandamide to suppress GABA release [134]. Additionally, this dual interaction has been shown to mediate the estradiol effects on hippocampal memory consolidation [135]. ERα-mGluR5 signaling was seen exclusively in female striatal neurons as well [136]. This interaction of receptors has not been investigated in rodent models of FXS. Future studies should evaluate this sex-specific mechanism in female neurons as it could provide an explanation for the sex differences seen in* Fmr1* KO mice.

## Conclusions

This is the first study to test and report sex differences during development in sensory processing in an ASD animal model. In terms of temporal processing, we used the 40 Hz ASSR paradigm, which models phonemic rates in speech [137]. Slower oscillations (delta to theta) may be more relevant to aspects of intonation and syllabic rates, and other aspects of speech with slower rates. Future studies will examine 10 and 20 Hz ASSRs in the *Fmr1* KO and WT mice, that may allow a prediction of the nature of speech deficits in humans with FXS. Given the robust sex differences and different trajectories of temporal processing versus hypersensitivity phenotypes in male and female mice with an identical gene knockout, future studies should examine possible role of gonadal hormones in the emergence of sex differences, either with gonadectomy at specific ages, or implants to release hormones over a specific time window. The peri-pubertal window is a critical period of development in *Fmr1* KO mice that is marked by cortical hyperexcitability and reduced inhibitory interneuron function [70, 75, 138]. However, these studies were carried out only in male mice. Future studies will characterize these developmental milestones in female *Fmr1* KO mice. In order to effectively treat humans with FXS, it is imperative to understand the sex differences and the developmental trajectory of phenotypes that are likely to be used as clinical outcome measures, as opposed to just adult male comparisons. The differing trajectories of temporal processing and hypersensitivity in female compared to male KO mice suggests that more developmental studies of human females with FXS are needed. Future studies in humans with FXS should evaluate temporal processing across age in both males and females to determine if similar delays in development are present, and if the delay relates to language function.

### Supplementary Information

**Additional file 1.** Full statistical analysis of female WT and KO gap-ASSR data. Three-way repeated measures ANOVA results for gap-ASSR analysis. Mauchly Tests for sphericity were utilized and *p*-values were corrected using the Greenhouse-Geisser method where necessary. See text for post hoc results. Bold text indicates statistical significance (*p* ≤ 0.05).

**Additional file 2.** Full statistical analysis of male and female KO gap-ASSR data. Two-way repeated measures ANOVA results for gap-ASSR analysis comparing male and female *Fmr1* KO mice. Sex differences are only seen at p30 in the FC. Degrees of freedom and *p*-values were corrected for lack of sphericity using the Greenhouse-Geisser method. Bold text indicates statistical significance (*p* ≤ 0.05).

**Additional file 3.** Full statistical analysis of male and female WT gap-ASSR data. Two-way repeated measures ANOVA results for gap-ASSR analysis comparing male and female WT mice. No sex differences were present at any age. Degrees of freedom and *p*-values were corrected for lack of sphericity using the Greenhouse-Geisser epsilon-hat method. Bold text indicates statistical significance (*p* ≤ 0.05).

**Additional file 4.** Full statistical analysis of female development ERP data. Two-way ANOVA results for ERP analysis. Post hoc comparisons were done using Tukey’s and Bonferroni’s multiple comparisons tests. See text for post hoc results. Bold text indicates statistical significance (*p* ≤ 0.05).

**Additional file 5. **Full statistical analysis of WT development ERP data. Two-way ANOVA results for ERP analysis. Post hoc comparisons were done using Tukey’s and Bonferroni’s multiple comparisons tests. See text for post hoc results. Bold text indicates statistical significance (*p* ≤ 0.05).

**Additional file 6. **Full statistical analysis of KO development ERP data. Two-way ANOVA results for ERP analysis. Post hoc comparisons were done using Tukey’s and Bonferroni’s multiple comparisons tests. See text for post hoc results. Bold text indicates statistical significance (*p* ≤ 0.05).

## Data Availability

The data that support the findings of this study are available from the corresponding author with reasonable request.

## Abbreviations

ASD: Autism spectrum disorders
KO: Knock-out
FXS: Fragile X syndrome
WT: Wild-type
ERP: Event related potentials
ASSR: Auditory steady state response
P: Postnatal
Fmr1: Fragile X messenger ribonucleoprotein gene
FMRP: Fragile X messenger ribonucleoprotein
AC: Auditory cortex
FC: Frontal cortex
i.p.: Intraperitoneal
STP: Single trial power
ITPC: Inter-trial phase clustering
