# Supplementary material for: Sex differences during development in cortical temporal processing and event related potentials in wild-type and fragile X syndrome model mice
Source: J Neurodev Disord. 2024 May 8;16:24. doi: 10.1186/s11689-024-09539-8 (PMC11077726; doi:10.1186/s11689-024-09539-8)
Supplement: Supplementary file 1 — Additional file 1. Full statistical analysis of female WT and KO gap-ASSR data. Three-way repeated measures ANOVA results for gap-ASSR analysis. Mauchly Tests for sphericity were utilized and p-values were corrected using the Greenhouse-Geisser method where necessary. See text for post hoc results. Bold text indicates statistical significance (p ≤ 0.05). [file 11689_2024_9539_MOESM1_ESM.pdf]

Additional File 1. Full statistical analysis of female WT and KO gap-ASSR data.

Cortical Region	Modulation Depth	Factor/Interaction	ANOVA Results	Adjusted p-value
AC	100%	Genotype	F(1,47)=0.9628	0.3315
		Age	F(2,47)=2.5722	0.0870
		**Gap Duration**	**F(5,235)=3.7481**	**0.0165**
		Genotype x Gap Duration	F(5,235)=0.4926	0.6642
		Age x Gap Duration	F(10,235)=1.2445	0.2913
		Genotype x Age	F(2,47)=1.9964	0.1471
		Genotype x Age x Gap Duration	F(10,235)=1.2189	0.3033
AC	75%	Genotype	F(1,47)=0.3658	0.5482
		Age	F(2,47)=2.1187	0.1315
		Gap Duration	F(5,235)=2.4164	0.0715
		Genotype x Gap Duration	F(5,235)=0.2511	0.8530
		Age x Gap Duration	F(10,235)=1.5942	0.1562
		Genotype x Age	F(2,47)=0.7530	0.4765
		Genotype x Age x Gap Duration	F(10,235)=0.3116	0.9250
FC	100%	**Genotype**	**F(1,47)=6.0378**	**0.01774**
		**Age**	**F(2,47)=3.6924**	**0.0324**
		Gap Duration	F(5,235)=1.3452	0.2644
		Genotype x Gap Duration	**F(5,235)=3.9951**	**0.0130**
		Age x Gap Duration	F(10,235)=1.9202	0.0935
		Genotype x Age	F(2,47)=1.9326	0.1560
		**Genotype x Age x Gap Duration**	**F(10,235)=2.5717**	**0.0286**
FC	75%	**Genotype**	**F(1,47)=6.0071**	**0.0180**
		**Age**	**F(2,47)=6.7788**	**0.0025**
		**Gap Duration**	**F(5,235)=4.6730**	**0.0037**
		Genotype x Gap Duration	F(5,235)=2.5335	0.0589
		**Age x Gap Duration**	**F(10,235)=4.0257**	**0.0009**
		Genotype x Age	F(2,47)=1.4508	0.2446
		Genotype x Age x Gap Duration	F(10,235)=1.0932	0.3694

Three-way repeated measures ANOVA results for gap-ASSR analysis. Mauchly Tests for sphericity were utilized and p-values were corrected using the Greenhouse-Geisser method where necessary. See text for post hoc results. Bold text indicates statistical significance ($p \leq 0.05$).
