# Supplementary material for: Sex differences during development in cortical temporal processing and event related potentials in wild-type and fragile X syndrome model mice
Source: J Neurodev Disord. 2024 May 8;16:24. doi: 10.1186/s11689-024-09539-8 (PMC11077726; doi:10.1186/s11689-024-09539-8)
Supplement: Supplementary file 2 — Additional file 2. Full statistical analysis of male and female KO gap-ASSR data. Two-way repeated measures ANOVA results for gap-ASSR analysis comparing male and female Fmr1 KO mice. Sex differences are only seen at p30 in the FC. Degrees of freedom and p-values were corrected for lack of sphericity using the Greenhouse-Geisser method. Bold text indicates statistical significance (p ≤ 0.05). [file 11689_2024_9539_MOESM2_ESM.pdf]

Additional File 2. Full statistical analysis of male and female KO gap-ASSR data.

**Age**	**Cortical Region**	**Modulation Depth**	**Factor/Interaction**	**ANOVA Results**	**Adjusted p-value**
P21	AC	100%	Interaction	F(5,80)=0.3211	0.8989
			**Gap Duration**	**F(2.708,43.34)=11.06**	**<0.0001**
			Sex	F(1,16)=0.02711	0.8713
P21	AC	75%	Interaction	F(5,80)=0.8956	0.4882
			**Gap Duration**	**F(3.233,51.73)=8.822**	**<0.0001**
			Sex	F(1,16)=0.0053	0.9424
P21	FC	100%	Interaction	F(5,80)=0.1754	0.9711
			**Gap Duration**	**F(3.740,59.84)=7.125**	**0.0001**
			Sex	F(1,16)=0.6813	0.4213
P21	FC	75%	Interaction	F(5,80)=0.5624	0.7285
			**Gap Duration**	**F(3.847,61.55)=16.67**	**<0.0001**
			Sex	F(1,16)=0.4218	0.5252
P30	AC	100%	Interaction	F(5,85)=1.187	0.3225
			**Gap Duration**	**F(2.628,44.67)=14.42**	**<0.0001**
			Sex	F(1,17)=2.700	0.1187
P30	AC	75%	Interaction	F(5,85)=0.8130	0.5436
			**Gap Duration**	**F(3.024,51.41)=9.528**	**<0.0001**
			Sex	F(1,17)=2.037	0.1716
P30	FC	100%	**Interaction**	**F(5,85)=3.911**	**0.0031**
			**Gap Duration**	**F(3.160,53.73)=26.73**	**<0.0001**
			**Sex**	**F(1,17)=7.150**	**0.0160**
P30	FC	75%	**Interaction**	**F(5,85)=3.967**	**0.0028**
			**Gap Duration**	**F(2.962,50.36)=21.45**	**<0.0001**
			**Sex**	**F(1,17)=11.53**	**0.0034**
P60	AC	100%	Interaction	**F(5,85)=0.2513**	0.9381
			**Gap Duration**	**F(2.405,40.88)=29.66**	**<0.0001**
			Sex	F(1,17)=0.1049	0.7500
P60	AC	75%	Interaction	**F(5,85)=0.1959**	0.9633
			**Gap Duration**	**F(2.455,41.73)=30.59**	**<0.0001**
			Sex	F(1,17)=0.2712	0.6092
P60	FC	100%	Interaction	F(5,85)=0.9318	0.4647
			**Gap Duration**	**F(2.910,49.46)=28.83**	**<0.0001**
			Sex	F(1,17)=0.0223	0.8831
P60	FC	75%	Interaction	F(5,85)=0.4046	0.8444
			**Gap Duration**	**F(3.577,60.81)=64.18**	**<0.0001**
			Sex	F(1,17)=0.2619	0.6154

Two-way repeated measures ANOVA results for gap-ASSR analysis comparing male and female Fmr1 KO mice. Sex differences are only seen at p30 in the FC. Degrees of freedom and p-values were corrected for lack of sphericity using the Greenhouse-Geisser method. Bold text indicates statistical significance ($p \leq 0.05$).
