# Supplementary material for: Sex differences during development in cortical temporal processing and event related potentials in wild-type and fragile X syndrome model mice
Source: J Neurodev Disord. 2024 May 8;16:24. doi: 10.1186/s11689-024-09539-8 (PMC11077726; doi:10.1186/s11689-024-09539-8)
Supplement: Supplementary file 3 — Additional file 3. Full statistical analysis of male and female WT gap-ASSR data. Two-way repeated measures ANOVA results for gap-ASSR analysis comparing male and female WT mice. No sex differences were present at any age. Degrees of freedom and p-values were corrected for lack of sphericity using the Greenhouse-Geisser epsilon-hat method. Bold text indicates statistical significance (p ≤ 0.05). [file 11689_2024_9539_MOESM3_ESM.pdf]

Additional File 3. Full statistical analysis of male and female WT gap-ASSR data.

Age	Cortical Region	Modulation Depth	Factor/Interaction	ANOVA Results	Adjusted p-value
P21	AC	100%	Interaction	F(5,95)=0.7051	0.6210
			**Gap Duration**	**F(1.855,32.25)=13.45**	**<0.0001**
			Sex	F(1,19)=3.482e-005	0.9954
P21	AC	75%	Interaction	F(5,95)=0.8763	0.5001
			**Gap Duration**	**F(2.294,43.58)=6.872**	**0.0017**
			Sex	F(1,19)=0.0296	0.8650
P21	FC	100%	Interaction	F(5,95)=0.2110	0.9571
			**Gap Duration**	**F(2.161,41.07)=21.59**	**<0.0001**
			Sex	F(1,19)=0.2328	0.6350
P21	FC	75%	Interaction	F(5,95)=0.7227	0.6080
			**Gap Duration**	**F(1.762,33.49)=29.02**	**<0.0001**
			Sex	F(1,19)=0.6102	0.4444
P30	AC	100%	Interaction	F(5,85)=1.923	0.0989
			**Gap Duration**	**F(4.066,69.12)=26.12**	**<0.0001**
			Sex	F(1,17)=0.6887	0.4181
P30	AC	75%	Interaction	F(5,85)=0.2505	0.9385
			**Gap Duration**	**F(2.749,46.74)=13.02**	**<0.0001**
			Sex	F(1,17)=0.0496	0.8264
P30	FC	100%	Interaction	F(5,85)=0.7688	0.5748
			**Gap Duration**	**F(3.748,63.72)=12.81**	**<0.0001**
			Sex	F(1,17)=0.5620	0.4637
P30	FC	75%	Interaction	F(5,85)=0.6334	0.6748
			**Gap Duration**	**F(3.708,63.04)=38.15**	**<0.0001**
			Sex	F(1,17)=0.3719	0.5500
P60	AC	100%	Interaction	F(5,85)=0.6398	0.6699
			**Gap Duration**	**F(2.576,43.80)=32.67**	**<0.0001**
			Sex	F(1,17)=0.6390	0.4351
P60	AC	75%	Interaction	F(5,85)=0.7737	0.5714
			**Gap Duration**	**F(2.294,39.00)=35.44**	**<0.0001**
			Sex	F(1,17)=0.2400	0.6304
P60	FC	100%	Interaction	F(5,85)=0.7762	0.5696
			**Gap Duration**	**F(3.919,66.63)=44.83**	**<0.0001**
			Sex	F(1,17)=0.1551	0.6986
P60	FC	75%	Interaction	F(5,85)=1.598	0.1694
			**Gap Duration**	**F(3.677,62.51)=76.88**	**<0.0001**
			Sex	F(1,17)=0.0253	0.8754

Two-way repeated measures ANOVA results for gap-ASSR analysis comparing male and female WT mice. No sex differences were present at any age. Degrees of freedom and p-values were corrected for lack of sphericity using the Greenhouse-Geisser epsilon-hat method. Bold text indicates statistical significance ($p \leq 0.05$).
