# Supplementary material for: Sex differences during development in cortical temporal processing and event related potentials in wild-type and fragile X syndrome model mice
Source: J Neurodev Disord. 2024 May 8;16:24. doi: 10.1186/s11689-024-09539-8 (PMC11077726; doi:10.1186/s11689-024-09539-8)
Supplement: Supplementary file 4 — Additional file 4. Full statistical analysis of female development ERP data. Two-way ANOVA results for ERP analysis. Post hoc comparisons were done using Tukey’s and Bonferroni’s multiple comparisons tests. See text for post hoc results. Bold text indicates statistical significance (p ≤ 0.05). [file 11689_2024_9539_MOESM4_ESM.pdf]

Additional File 4. Full statistical analysis of female development ERP data.

Cortical Region	ERP Component	Factor	ANOVA Results	p-value
AC	P1 Amplitude:	**Interaction**	**F(2,47)=3.179**	**0.0507**
		**Age**	**F(2,47)=12.03**	**<0.0001**
		**Genotype**	**F(1,47)=12.39**	**0.0010**
	N1 Amplitude:	Interaction	F(2,47)=1.292	0.2843
		**Age**	**F(2,47)=12.50**	**<0.0001**
		**Genotype**	**F(1,47)=13.94**	**0.0005**
	P2 Amplitude:	Interaction	F(2,47)=2.022	0.1438
		**Age**	**F(2,47)=3.960**	**0.0257**
		**Genotype**	**F(1,47)=8.327**	**0.0059**
FC	P1 Amplitude:	**Interaction**	**F(2,47)=3.713**	**0.0318**
		**Age**	**F(2,47)=7.045**	**0.0021**
		Genotype	F(1,47)=1.562	0.2175
	N1 Amplitude:	**Interaction**	**F(2,47)=4.987**	**0.0109**
		**Age**	**F(2,47)=14.40**	**<0.0001**
		**Genotype**	**F(1,47)=11.60**	**0.0014**
	P2 Amplitude:	Interaction	F(2,47)=0.8274	0.4434
		**Age**	**F(2,47)=3.851**	**0.0283**
		Genotype	F(1,47)=0.1218	0.7287
AC	P1 Latency:	Interaction	F(2,47)=2.187	0.1235
		Age	F(2,47)=1.931	0.1564
		Genotype	F(1,47)=0.6171	0.4361
	N1 Latency:	Interaction	F(2,47)=2.009	0.1455
		**Age**	**F(2,47)=5.729**	**0.0059**
		Genotype	F(1,47)=0.0706	0.7916
	P2 Latency:	Interaction	F(2,47)=1.445	0.2461
		Age	F(2,47)=0.4215	0.6585
		Genotype	F(1,47)=2.342	0.1327
FC	P1 Latency:	Interaction	F(2,47)=0.0354	0.9643
		Age	F(2,47)=2.823	0.0695
		Genotype	F(1,47)=0.9239	0.3414
	N1 Latency:	Interaction	F(2,47)=0.8245	0.4447
		**Age**	**F(2,47)=7.105**	**0.0020**
		Genotype	F(1,47)=1.141	0.2909
	P2 Latency:	**Interaction**	**F(2,47)=11.18**	**0.0001**
		**Age**	**F(2,47)=3.555**	**0.0365**
		Genotype	F(1,47)=1.494	0.2277

Two-way ANOVA results for ERP analysis. Post hoc comparisons were done using Tukey's and Bonferroni's multiple comparisons tests. See text for post hoc results. Bold text indicates statistical significance ($p \leq 0.05$).
