# Supplementary material for: Sex differences during development in cortical temporal processing and event related potentials in wild-type and fragile X syndrome model mice
Source: J Neurodev Disord. 2024 May 8;16:24. doi: 10.1186/s11689-024-09539-8 (PMC11077726; doi:10.1186/s11689-024-09539-8)
Supplement: Supplementary file 5 — Additional file 5. Full statistical analysis of WT development ERP data. Two-way ANOVA results for ERP analysis. Post hoc comparisons were done using Tukey’s and Bonferroni’s multiple comparisons tests. See text for post hoc results. Bold text indicates statistical significance (p ≤ 0.05). [file 11689_2024_9539_MOESM5_ESM.pdf]

Additional File 5. Full statistical analysis of WT development ERP data.

Cortical Region	ERP Component	Factor	ANOVA Results	p-value
AC	P1 Amplitude:	Interaction	F(2,53)=2.202	0.1206
		Age	F(2,53)=1.729	0.1873
		Sex	F(1,53)=0.0689	0.7939
	N1 Amplitude:	Interaction	F(2,53)=0.8576	0.4300
		**Age**	**F(2,53)=4.289**	**0.0188**
		Sex	F(1,53)=0.9495	0.3343
	P2 Amplitude:	Interaction	F(2,53)=0.3466	0.7087
		**Age**	**F(2,53)=3.895**	**0.0264**
		Sex	F(1,53)=0.1412	0.7086
FC	P1 Amplitude:	Interaction	F(2,53)=1.460	0.2415
		Age	F(2,53)=0.0029	0.9971
		Sex	F(1,53)=1.067	0.3062
	N1 Amplitude:	Interaction	F(2,53)=2.609	0.0830
		Age	F(2,53)=1.770	0.1803
		Sex	F(1,53)=0.6039	0.4405
	P2 Amplitude:	Interaction	F(2,53)=2.569	0.0862
		Age	F(2,53)=2.502	0.0916
		Sex	F(1,53)=0.8089	0.3725
AC	P1 Latency:	Interaction	F(2,53)=1.142	0.3268
		Age	F(2,53)=1.935	0.1544
		Sex	F(1,53)=2.848	0.0974
	N1 Latency:	Interaction	F(2,53)=2.322	0.1080
		**Age**	**F(2,53)=9.198**	**0.0004**
		Sex	F(1,53)=0.2755	0.6018
	P2 Latency:	Interaction	F(2,53)=0.6371	0.5328
		Age	F(2,53)=3.130	0.0519
		Sex	F(1,53)=2.747	0.1034
FC	P1 Latency:	Interaction	F(2,53)=0.078	0.9250
		Age	F(2,53)=1.919	0.1568
		Sex	F(1,53)=0.6243	0.4330
	N1 Latency:	Interaction	F(2,53)=1.279	0.2867
		**Age**	**F(2,53)=9.972**	**0.0002**
		Sex	F(1,53)=0.7294	0.3969
	P2 Latency:	Interaction	F(2,53)=0.4331	0.6508
		Age	F(2,53)=1.793	0.1764
		**Sex**	**F(1,53)=11.90**	**0.0011**

Two-way ANOVA results for ERP analysis. Post hoc comparisons were done using Tukey's and Bonferroni's multiple comparisons tests. See text for post hoc results. Bold text indicates statistical significance ($p \leq 0.05$).
