# Supplementary material for: Sex differences during development in cortical temporal processing and event related potentials in wild-type and fragile X syndrome model mice
Source: J Neurodev Disord. 2024 May 8;16:24. doi: 10.1186/s11689-024-09539-8 (PMC11077726; doi:10.1186/s11689-024-09539-8)
Supplement: Supplementary file 6 — Additional file 6. Full statistical analysis of KO development ERP data. Two-way ANOVA results for ERP analysis. Post hoc comparisons were done using Tukey’s and Bonferroni’s multiple comparisons tests. See text for post hoc results. Bold text indicates statistical significance (p ≤ 0.05). [file 11689_2024_9539_MOESM6_ESM.pdf]

Additional File 6. Full statistical analysis of KO development ERP data.

Cortical Region	ERP Component	Factor	ANOVA Results	p-value
AC	P1 Amplitude:	Interaction	F(2,50)=0.7898	0.4595
		**Age**	**F(2,50)=21.28**	**<0.0001**
		Sex	F(1,50)=0.9544	0.3333
	N1 Amplitude:	Interaction	F(2,50)=2.486	0.0935
		**Age**	**F(2,50)=7.916**	**0.0010**
		Sex	F(1,50)=1.747	0.1922
	P2 Amplitude:	**Interaction**	**F(2,50)=3.825**	**0.0285**
		Age	F(2,50)1.903	0.1598
		**Sex**	**F(1,50)=4.555**	**0.0378**
FC	P1 Amplitude:	Interaction	F(2,50)=1.002	0.3743
		**Age**	**F(2,50)=10.83**	**0.0001**
		Sex	F(1,50)=0.3887	0.5358
	N1 Amplitude:	**Interaction**	**F(2,50)=6.737**	**0.0026**
		**Age**	**F(2,50)=16.96**	**<0.0001**
		**Sex**	**F(1,50)=4.119**	**0.0478**
	P2 Amplitude:	**Interaction**	**F(2,50)=3.507**	**0.0376**
		Age	F(2,50)=0.8073	0.4518
		**Sex**	**F(1,50)=9.931**	**0.0027**
AC	P1 Latency:	Interaction	F(2,50)=2.242	0.1168
		**Age**	**F(2,50)=5.773**	**0.0055**
		Sex	F(1,50)=0.0156	0.9008
	N1 Latency:	Interaction	F(2,50)=0.2806	0.7565
		Age	F(2,50)=1.695	0.1940
		Sex	F(1,50)=0.0838	0.7734
	P2 Latency:	Interaction	F(2,50)=0.7940	0.4576
		Age	F(2,50)=0.4282	0.6540
		Sex	F(1,50)=0.0013	0.9714
FC	P1 Latency:	Interaction	F(2,50)=0.5262	0.5941
		Age	F(2,50)=3.034	0.0570
		Sex	F(1,50)=0.1518	0.6984
	N1 Latency:	Interaction	F(2,50)=0.8430	0.4364
		Age	F(2,50)=1.986	0.1479
		Sex	F(1,50)=0.1684	0.6833
	P2 Latency:	**Interaction**	**F(2,50)=3.872**	**0.0273**
		**Age**	**F(2,50)=9.160**	**0.0004**
		Sex	F(1,50)=2.031	0.1603

Two-way ANOVA results for ERP analysis. Post hoc comparisons were done using Tukey's and Bonferroni's multiple comparisons tests. See text for post hoc results. Bold text indicates statistical significance ($p \leq 0.05$).
